# Electrostatic Interaction with the Bacterial Cell Envelope Tunes the Lytic Activity of Two Novel Peptidoglycan Hydrolases

**DOI:** 10.1128/spectrum.00455-22

**Published:** 2022-04-25

**Authors:** Alicja Wysocka, Łukasz Łężniak, Elżbieta Jagielska, Izabela Sabała

**Affiliations:** a International Institute of Molecular and Cell Biology in Warsaw, Warsaw, Poland; b Mossakowski Medical Research Institute Polish Academy of Sciences, Warsaw, Poland; University of Manitoba

**Keywords:** peptidoglycan, hydrolase, electrostatics, *Staphylococcus aureus*, cell wall, enzybiotics, net charge, *Staphylococcus*

## Abstract

Peptidoglycan (PG) hydrolases, due to their crucial role in the metabolism of the bacterial cell wall (CW), are increasingly being considered suitable targets for therapies, and a potent alternative to conventional antibiotics. In the light of contradictory data reported, detailed mechanism of regulation of enzymes activity based on electrostatic interactions between hydrolase molecule and bacterial CW surface remains unknown. Here, we report a comprehensive study on this phenomenon using as a model two novel PG hydrolases, SpM23_A, and SpM23_B, which although share the same bacterial host, similarities in sequence conservation, domain architecture, and structure, display surprisingly distinct net charges (in 2D electrophoresis, pI 6.8, and pI 9.7, respectively). We demonstrate a strong correlation between hydrolases surface net charge and the enzymes activity by modulating the charge of both, enzyme molecule and bacterial cell surface. Teichoic acids, anionic polymers present in the bacterial CW, are shown to be involved in the mechanism of enzymes activity regulation by the electrostatics-based interplay between charged bacterial envelope and PG hydrolases. These data serve as a hint for the future development of chimeric PG hydrolases of desired antimicrobial specificity.

**IMPORTANCE** This study shows direct relationship between the surface charge of two recently described enzymes, SpM23_A and SpM23_B, and bacterial cell walls. We demonstrate that by (i) surface charge probing of bacterial strains collection, (ii) reduction of the net charge of the positively charged enzyme, and (iii) altering the net charge of the bacterial surface by modifying the content and composition of teichoic acids. In all cases, we observed that lytic activity and binding strength of SpM23 enzymes, are regulated by electrostatic interactions with the bacterial cell envelope and that this interaction contributes to the determination of the spectrum of susceptible bacterial species. Moreover, we revealed the regulatory role of charged cell wall components, namely, teichoic and lipoteichoic acids, over the SpM23 enzymes. We believe that our findings make an important contribution to understand the means of hydrolases activity regulation in the complex environment of the bacterial cell wall.

## INTRODUCTION

**FIG 1:**
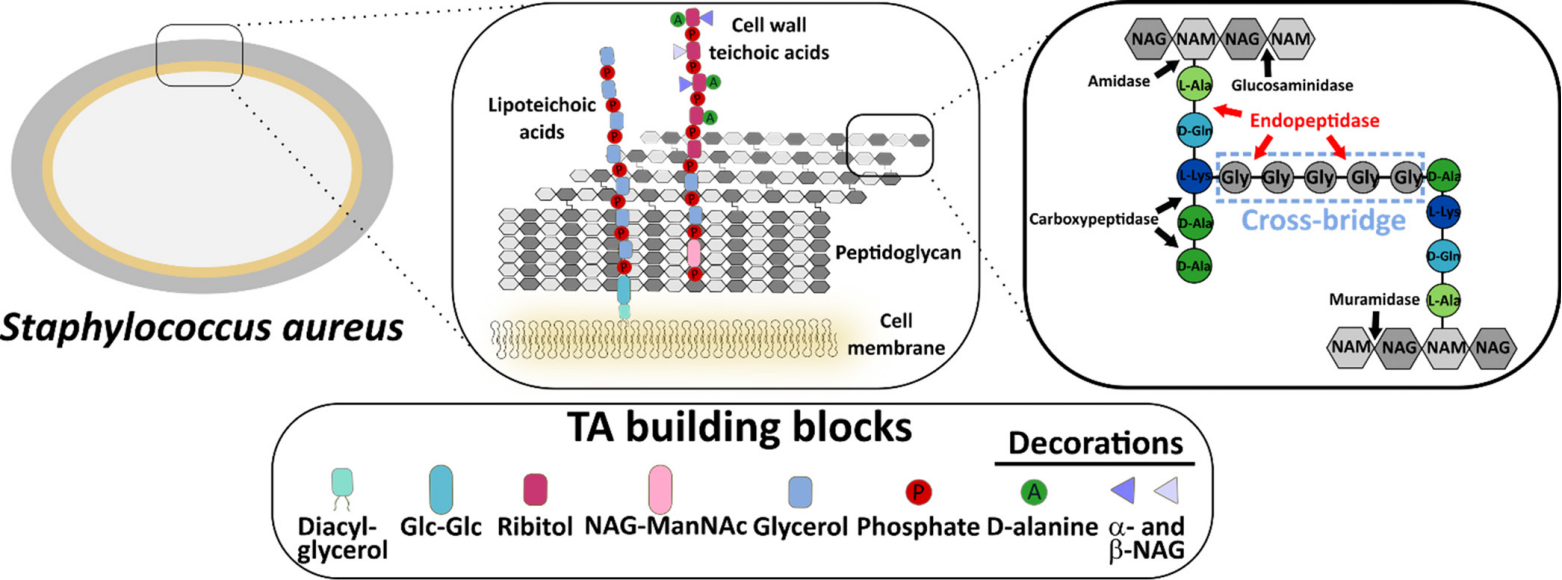
Schematic representation of the CW composition and cleavage sites for hydrolytic enzymes in Staphylococcus aureus. The arrows indicate cleavage sites of selected hydrolytic enzymes involved in the PG cleavage. NAG, *N*-Acetylglucosamine; NAM, *N*-Acetylmuramic acid; ManNAc, *N*-Acetylmannosamine; Glc, glucose. Based on Vollmer et al. (18), Santa Maria et al. (19). The predicted cleavage site determined for M23 family (endo)peptidases is highlighted in red.

The cell wall (CW) of Gram-positive bacteria has a multicomponent and inhomogeneous nature. Due to its outer localization, the bacterial CW is the site of many interactions with the external environment. From a mechanistic point of view, peptidoglycan (PG), the main component of the CW, plays a key role for maintaining its integrity. Composed of a polysaccharide polymer featuring repeated units of *N*-Acetylglucosamine (NAG) and *N*-Acetylmuramic acid (NAM), cross-linked by short peptides, PG guarantees the necessary elasticity of the cell upon its growth, elongation, and division; and also withstands high internal turgor (1) (Fig. 1). Teichoic acids, composed of anionic chains formed by repeated phosphodiester units, are the most abundant element of CW, and constitute 30% to 60% of the mass of the CW of Gram-positive bacteria (2).

PG is a substrate for many hydrolytic enzymes, which target it in the course of the bacterial cell development (Fig. 1). The protein surface charge is an important factor for the interactions between the PG hydrolases and the bacterial CW (3–5). A current hypothesis suggests that the positive net surface charge of PG hydrolases allows them to interact with the negatively charged bacterial (5, 6) or eukaryotic cells surfaces (7, 8); however, other reports have undermined this theory (9). This issue has been investigated by many research groups and discussed in several reports but their conclusions have been inconsistent, probably because the proteins analyzed were different in other respects (i.e., distribution of the surface charge, electrostatics of the binding groove) except for the net surface charge or because the studied enzymes were artificially engineered (9).

We addressed the discrepancies in this field by taking advantage of SpM23_A and SpM23_B, two peptidoglycan hydrolases produced by Staphylococcus pettenkoferi (10). We recently reported the discovery of these two novel PG hydrolases, which display prominent sequence conservation (60% of amino acid sequence identity), same modular architecture (inhibitory region, catalytic and binding domain), and conservation of catalytic and binding motifs. We observed that they both bind and hydrolase *S. aureus* cell wall, that make them good candidates for development of new effective treatments targeting this pathogen.

Overall, SpM23_A and SpM23_B resemble thoroughly studied M23 peptidase, lysostaphin, which targets *S. aureus* pentaglycine bridge (4) (Fig. 1). Several other hydrolases from M23 family with similar catalytic domains have been described so far, i.e., LytM and LytU, which predominantly cleave glycine–glycine bonds in the PG cross-bridges characteristic for Staphylococcus species (11–13), zoocinA or EnpA, which hydrolyze glycine/l-alanine-d-alanine bonds in the PG cross-links present in Streptococcus and *Enterococcus*, respectively (14–16) or LasA, which has broader range of the cleavable substrates (glycine-glycine/alanine/phenylalanine, tyrosine/leucine) (17).

Apart from many similarities, SpM23_A and SpM23_B differ in their gene distribution (*spm23_A* is widely conserved, whereas *spm23_B* is rare), putative biological role (autolysin versus bacteriocin), and most interestingly, in their isoelectric points (10). The latter make them a perfect model for studying the effects of the electrostatic interactions between enzyme and substrate in reaction performed in the complex environment of bacterial CW.

Using a set of biochemical assays, we were able to identify the influence of the bacterial surface charge and the individual components of the CW, such as teichoic acids, on the activity and/or specificity of the PG hydrolases.

## RESULTS

### Two novel M23 peptidases display sequence and domain architecture similarities but opposite surface net charge.

**FIG 2:**
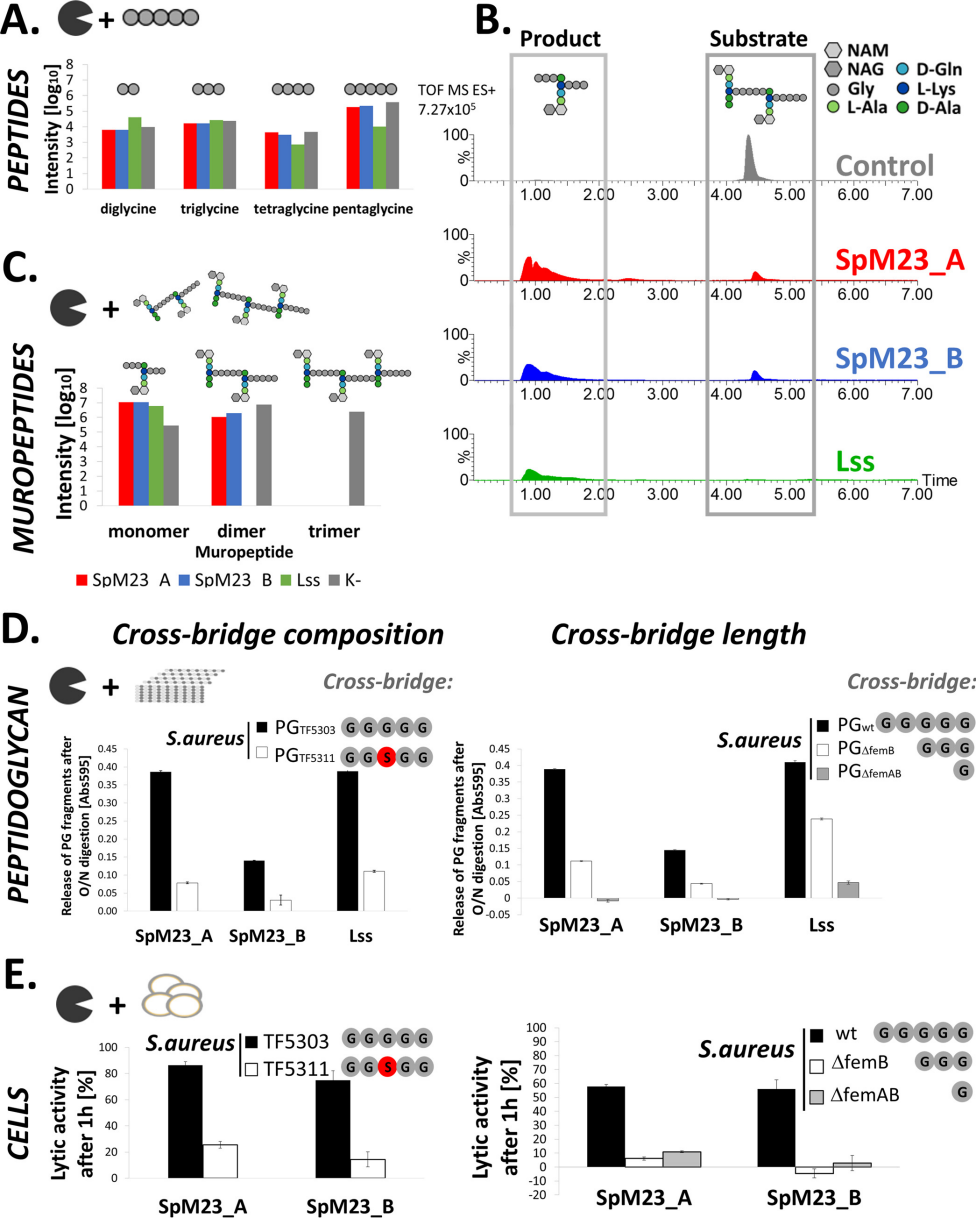
SpM23 enzymes target preferentially pentaglycine cross-bridge. (A) Digestion of the pentaglycine. Bars represents intensities of the peaks assigned to the masses corresponding to the di-, tri-, tetra-, and pentaglycine, that were identified in mass spectrometry analysis. The reaction was conducted at 50 mM glycine pH 8.0, 100 mM NaCl buffer for 24 h at 37°C with 4 μM enzymes and 2 mM pentaglycine. (B) Digestion of the muropeptides. The substrate was generated upon overnight mutanolysin treatment of PG of *S. aureus* NCTC 8325-4 in 50 mM glycine pH 8.0, 100 mM NaCl. Then, solubilized PG was mixed with enzymes (4 μM final concentration) and incubated for 24 h at 37°C. Ionogram demonstrates peaks assigned to muropeptide monomer (product) and dimer (substrate). NAG, *N*-Acetylglucosamine; NAM, *N*-Acetylmuramic acid. (C) Bars represent intensities of the peaks assigned to the masses corresponding to the muropeptide mono-, di-, and trimer that were identified in mass spectrometry analysis after digestion of the muropeptides by the SpM23 enzymes and Lss. The figure presents results from the same assay as described in panel B. (D) PG digestion of the SpM23 enzymes against *S. aureus* with altered cross-bridge composition and length. Activity of enzymes against purified PG from *S. aureus* TF5303 wild-type and TF5311 cross-bridge mutant (serine insertion) and *S. aureus* 8325 wild-type, *femB* and *femAB* deletion mutants (three and one glycine cross-bridge, respectively). The chart shows release of soluble fragments of PG stained with Remazol Brilliant Blue R after overnight incubation with 100 nM enzymes at 37°C in 50 mM glycine pH 8.0, 100 mM NaCl buffer. (E) Lytic assay against bacterial cells corresponding to that presented at the panel D. Lytic activity assay is indicated with chart bars and the average for each enzyme. The assay was performed in 50 mM glycine pH 8.0 buffer. The chart shows bacterial suspension turbidity reduction after 1 h incubation with 100 nM enzymes at room temperature (RT). Result are presented as a % of reduction of initial OD_600_.

We have previously reported that SpM23_A and SpM23_B cleave peptidoglycan of staphylococci and based on the similarities to lysostaphin, concluded that both enzymes target pentaglycine cross-bridges (10). To confirm that, we have probed their activity against pentaglycine, but failed to observe any digestion products for SpM23 enzymes (Fig. 2A). Assuming that pentaglycine might be too short of a pentapeptide substrate to be able to generate stable complex with enzymes, we decided to use muropeptides, obtained upon solubilization of the *S. aureus* NCTC 8325-4 PG by mutanolysin, instead. Obtained digestion profiles for SpM23 enzymes were the same as for lysostaphin, which was used as a positive control, proving that SpM23 enzymes are peptidoglycan hydrolases that cleave pentaglycine cross-bridges (monomers, dimers and trimers detected) (Fig. 2B and C).

Furthermore, we looked whether these enzymes could digest more complex substrates: cross-bridges of an altered composition or length. Purified PG from S. aureus and a set of its mutants with Gly to Ser substitution in the cross-bridge (TF5311 mutant; [32]) and cross-bridges shortened to three and one glycine (*S. aureus* 8325 Δ*femB* and Δ*femAB;* [33]) were subjected to digestion by SpM23 enzymes. Remazol staining results confirmed that both enzymes can digest S. aureus peptidoglycans. Moreover, altered cross-bridge composition and length impaired the activity of both SpM23 enzymes (Fig. 2D). Similar results were obtained when we tested enzymes lytic activity against whole staphylococcal cells. We could observe very effective cell lysis in the case of the WT S. aureus while serine insertion into cross-bridge diminished the lytic activity of SpM23_A and SpM23_B by 70% and 81%, respectively (Fig. 2E). The effect of length diminishing was even more detrimental on their activity, resulting in 89% loss of activity for SpM23_A and complete shut-down of SpM23_B lytic activity, while the cross-bridge was composed of three glycine. We observed 81% and 95% loss of initial activity of SpM23_A and spM23_B, respectively, while cross-bridge constitutes of single glycine (Fig. 2E). We concluded that SpM23_A and SpM23_B target *staphylococcal* cross-bridges preferentially composed of pentaglycine.

**FIG 3:**
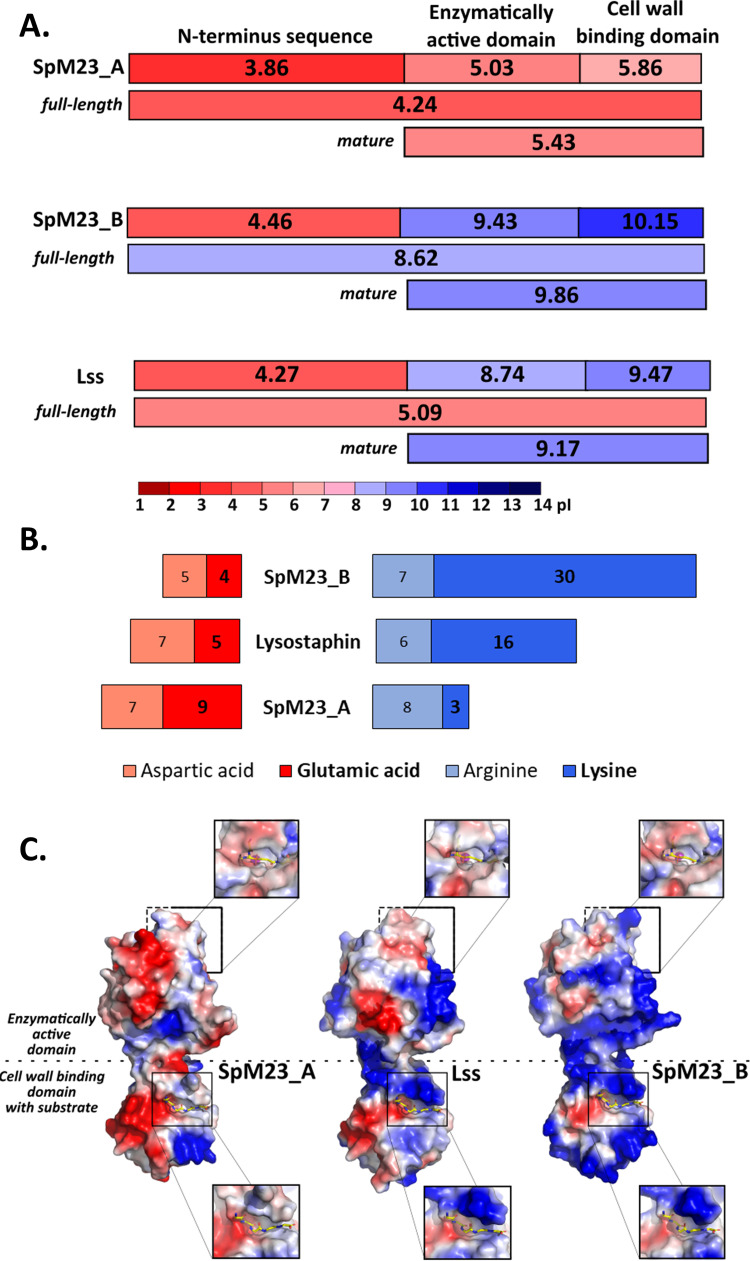
Modular architecture and charge analyses of the novel M23 peptidases. (A) Modular organization of SpM23_A, SpM23_B, and Lss and the pI values for each domain (GenBank Accession numbers: SpM23_A: WP_002472647.1, SpM23_B: ASE36562.1A, Lss: AAB53783.1). The theoretical pI was calculated with an isoelectric point calculator (34). (B) The charged amino acids present in the SpM23_A, Lss, and SpM23_B. The numbers in the boxes represent the number of negatively (red) and positively (blue) charged selected amino acids in the mature enzyme form (EAD and CBD domains). The predominant negatively charged amino acid in SpM23_A and the positively charged amino acid in SpM23_B are marked in bold. (C) Net charge distribution on the surface of the studied M23 enzymes. The analyses were performed in PyMol based on the models obtained in SWISS-MODEL server (35). Substrate and ligand binding grooves are presented for each of the enzymes in the zoomed panels. The surface of the molecules is coloured accordingly to its electrostatic potential, scaled from −50 (red, negative) to +50 kT/e (blue, positive).

Apart from many similarities, SpM23_A and SpM23_B differ in their isoelectric points (5.7 for SpM23_A and 10.3 for SpM23_B). Net charge discrepancies are observed for both the full-length and mature forms of the enzymes, but also while comparing each domain separately: enzymatically active domain (EAD) or CW binding domain (CBD, Fig. 3A, Fig. S1A). Hence, they constitute a perfect model for studying the role of the surface charge in regulation of specificity and activity of PG hydrolases.

We have analyzed the distribution of charged amino acids in SpM23_A and SpM23_B sequences and compared it with the distribution of corresponding amino acids within Lss sequence, which is the best characterized member of M23 family of peptidases with the intermediate values of pI (9.6). The majority of charged amino acids in SpM23_A and SpM23_B are located in the CBDs (Fig. S1B). Interestingly, the N-terminal propeptide in all analyzed cases is acidic and possesses the lowest pI of all domains.

Sequence analysis revealed that acidic amino acids outnumber basic ones in SpM23_A, whereas lysine is the most prominent charged amino acid in SpM23_B sequence (Fig. 3B). To illustrate the distribution of the charge on the enzyme’s surface, we generated models of the enzymes in the SWISS-MODELER server and subjected them to electrostatic charge distribution analyses, performed in PyMol (Fig. 3C). The results indicated clear differences in the charge distribution, with only a few spots of relatively conserved regions, such as in the active center and the substrate groove of the catalytic domains. More pronounced differences were seen in the ligand binding groove of the CBD; one side of the SpM23_A binding groove was neutral while in SpM23_B and Lss this region was highly positive. Other differences in charge distribution were also observed in other regions of the proteins; however, they have not been demonstrated to be directly involved in protein–CW interactions.

### The lytic specificities of SpM23_A and SpM23_B differ.

To understand the contribution of both, catalytic and CBD, domains to the overall specificity determination, we performed a lytic activity assay of the mature enzymes, consisting of both domains, and the binding assays for separated CBD domains.

**FIG 4:**
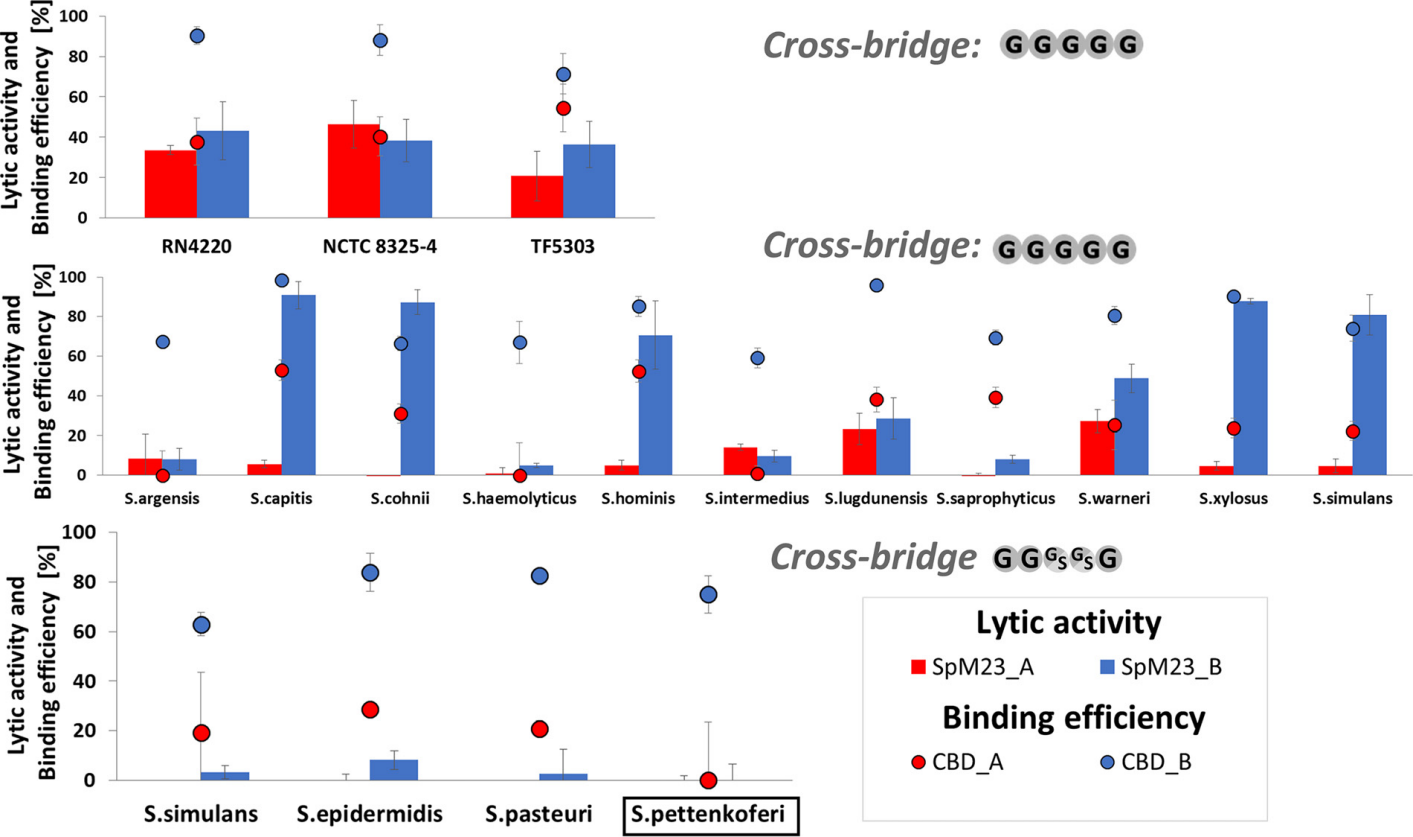
The lytic activity and binding spectra of the SpM23_A and SpM23_B differ. The results of the lytic activity assay are indicated with chart bars. The lytic activity was tested in a turbidity reaction assay performed in 50 mM glycine pH 8.0 and 100 mM NaCl buffer in the presence of 100 nM enzymes for 1 h at room temperature (RT). The experimental conditions are the same as used for the assays in Fig. 2E. The strain in which *m23_a* and *m23_b* genes were identified, namely, *S. pettenkoferi* VCU012, is framed. Cross-bridge amino acid composition was indicated in gray in single letter code. G/S, position in the cross-bridge occupied by glycine or serine. The binding of CBD_A and CBD_B are shown as dots. The bacterial cells were mixed with 1 μM each fluorescently labeled CBD in PBS buffer pH 7.2, incubated at RT. The cells were pelleted, and the fluorescence of the unbound fraction in the supernatant was measured; the results were normalized to the fluorescence of negative control (GFP only). The results are presented as percentages of reduced fluorescence after the addition of the tested CBDs. Schematic representation of the cross-bridge composition has been presented in the polarity from diamino acid side chain to D-Ala (listed as well in Table S1A). The error bars represent the standard deviation, calculated in three independent assays.

We tested the lytic activity of the mature forms of SpM23_A and SpM23_B on a range of bacterial species displaying variability in cross-bridges length and composition (23 species, Table S1A). None of the enzymes was active against Gram-negative bacteria while among Gram-positive species only staphylococci were lysed (Fig. 4, Fig. S2). Out of 15 staphylococcal species tested, eight were lysed by at least one SpM23 enzyme (arbitrary assumed as reduction of OD_600_ by at least 20% after 1 h). All susceptible species were also lysed by lysostaphin, known for its selectivity for the pentaglycine cross-bridge (Fig. S3) (36). Based on that, we concluded that only species with pentaglycine were susceptible to lysis by SpM23 enzymes, while those reported to contain serine in their cross-bridges were not. Interestingly, we noticed some exceptions to this rule; *S. argensis*, *S. haemolyticus*, S. intermedius, and *S. saprophyticus w*ere lysed by lysostaphin but not by any of our new enzymes. One possible reason for these discrepancies could be due to different extracellular polysaccharides content on the surface of various staphylococcal species and strains. We have therefore tested production of extracellular polyssacharides by plating bacteria on the BHI-Congo red medium (37) (Fig. S4A) We observed great variety of exopolysaccharides on the surfaces of tested strains but did not find any correlation between their production and the susceptibility pattern to the SpM23 enzymes. Furthermore, isolated PG from those strains appeared to be less prone to the action of the SpM23 enzymes compared with Lss (Fig. S4B). Therefore, we concluded that PG composition, including cross-bridge length and its content, affects the activity of SpM23 enzymes. However, it cannot be excluded that other CW components may also contribute to susceptibility toward the SpM23 enzymes.

We have noticed that the susceptibility of certain species toward one of the enzymes indicated the opposite toward the other: if a certain strain was efficiently lysed by one of the enzymes, at the same time, it was rather resistant to the activity of the other (Fig. S5). Moreover, the optimal lytic performance of SpM23_A was limited to low ionic strength conditions, whereas the SpM23_B was active in both low and high ionic strength buffers (Fig. S5).

Taking into account the discrepancies in the net charge of each enzyme, one might expect that the lytic activity would depend on the pH of the buffer, and the activity of SpM23_A would be shifted toward the buffers of low pH and inversely, SpM23_B should be more active in buffers with high pH. We ran a lytic activity assay in a range of pH values, but such relationship was not observed: SpM23_A was active at pH 6–8 and SpM23_B was active at pH 5 to 8 (Fig. S6).

### Binding spectrum does not explain lytic selectivity of the enzymes.

We wondered whether the observed differences in the activity of the investigated enzymes were determined by their binding efficiency. Binding assays with GFP-fused binding domains demonstrated that CBD_B attaches to all tested bacterial strains, including Gram-negative species, such as E. coli, even those against which SpM23_B does not display any lytic activity. Fluorescent imaging confirmed that CBD_B binds the outer surfaces of S. aureus and E. coli (Fig. S7). By contrast, CBD_A was much more selective; in general, it attached to the bacterial species that were also its lytic substrates (Fig. 4), but some exceptions from this rule was also observed. CBD_A could bind *S. capitis* and S. hominis, although its lytic activity against these two strains is limited (Fig. S5).

**FIG 5:**
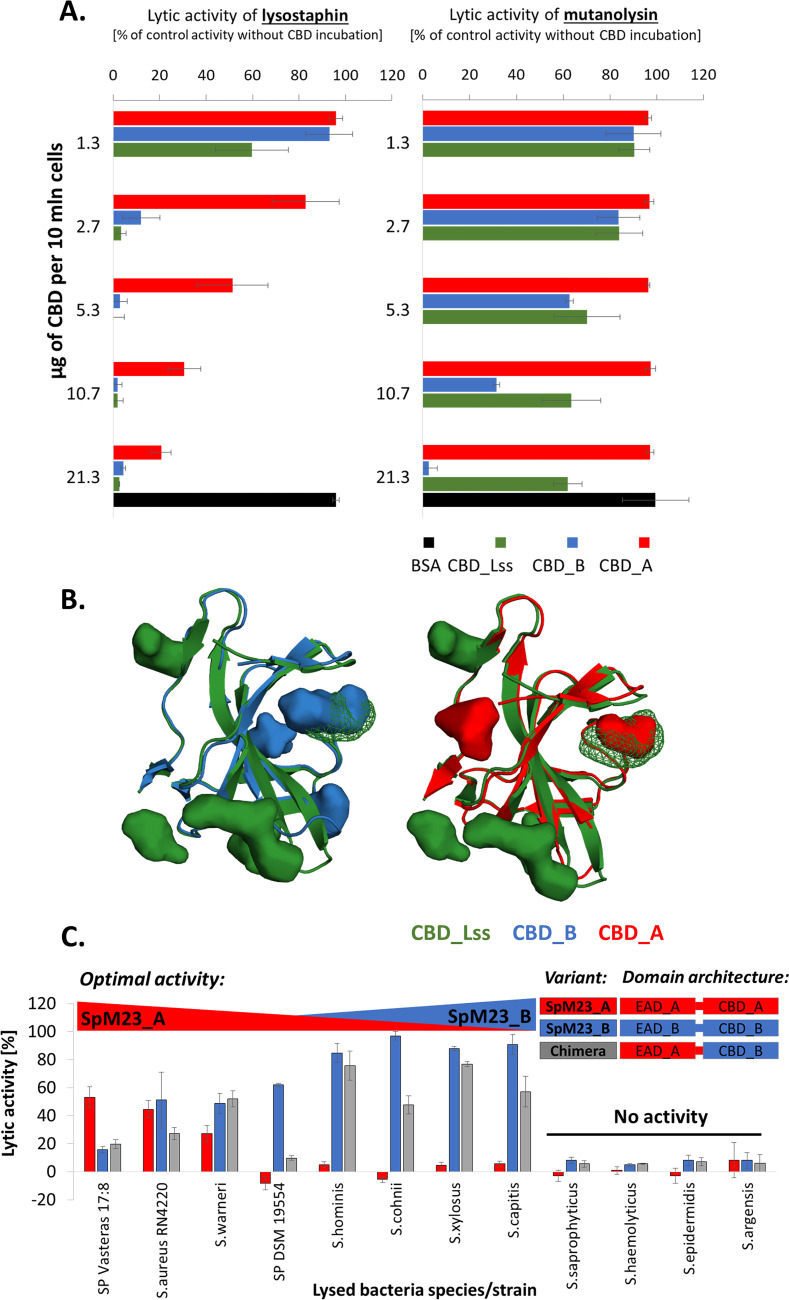
Binding domains analyses. (A) Competitive assay of the CBDs with Lss (left panel) and mutanolysin (right panel). Lss and mutanolysin activities are presented as the percentages of bacterial suspension turbidity reduction after 1 h of incubation with 100 nM Lss at room temperature (RT) or after 2 h of incubation of 100 U of mutanolysin at 37°C calculated as a percentage of the turbidity reduction in samples incubated without added CBD subtracted to the values of negative control (see Materials and Methods). The error bars represent the standard deviation, calculated in three independent assays. BSA was used as a negative control to identify the unspecific binding in the studied reaction conditions. (B) Binding pockets of CBD_A (indicated in red), CBD_B (blue), and CBD_Lss (green, PDB ID: 5LEO). The models for CBD_A and CBD_B were generated using the AlphaFold2 protein structure predictor (38), which scored both models as having high confidence (pLDDT > 90 for each chain). The binding pockets were generated using the POCASA tool (39). For both the structure and the pockets modeling, the parameters were set as default. All CBD domains were depicted in the cartoon representation. The volume of each pocket was marked as spheres colored accordingly. The binding groove previously defined for CBD_Lss (26) was indicated in the perforated type of surface. (C) Lytic activity for SpM23_A, SpM23_B, and chimeric enzyme. The lytic activity is presented as percentages or reductions of the bacterial suspension turbidity after 1 h of incubation with 100 nM enzymes at RT. The decrease in the turbidity of the bacteria suspension in the reaction buffer without the addition of the lytic enzyme was treated as a negative control and was subtracted from the presented values. SP, *S*. *pettenkoferi*. Schematic representation of the naturally occurring and artificially designed chimeric enzymes were presented in block representation on the chart. The error bars show the standard deviations calculated in three independent assays.

The broad spectrum of binding of the CBD_B domain, covering even Gram-negative species, suggests more general nature of these interactions. We sought to determine whether this domain binds pentaglycine cross-bridge or/and other elements of PG. The activity of PG hydrolases can be blocked by the binding of the CBD of another enzyme at the exact site or in close proximity to the target PG fragment, which serves as the enzyme binding ligand or/and substrate (23). When bacteria are incubated with increasing concentrations of CBD domains, their binding sites for the enzyme are pre-occupied, and the access of PG to the active site of the EAD domain is blocked. We have run ligand competition assays using CBDs isolated from SpM23 peptidases and Lss, which cleave pentaglycine cross-bridges, or mutanolysin (EC 3.2.1.17), which cleaves the bonds between NAM and NAG. The lytic test with Lss was performed on S. aureus NCTC 8325-4 as a representative of the Staphylococcus-type PG with pentaglycine cross-bridge, whereas the mutanolysin assay on the Streptococcus agalactiae strain, which does not contain polyglycine cross-bridge, and is susceptible to this lytic enzyme. We observed that CBD_B blocked the lytic activity of both Lss and mutanolysin (Fig. 5A), while CBD_A binding affected only Lss activity. Effect was also observed at relatively high concentrations of CBD_A compared with the inhibitory effects of CBD_Lss (26, 27). We supported our conclusions by performing the same assay using purified PG from *S. aureus* and *S. agalactiae* as a substrate and observed the same trend (Fig. S8). It need to be noted, that excess of CBD_A exhibits some inhibitory effect on mutanolysin digestion of PG as well (reduction by 44%). These results indicate that CBD_B binding restricts the access to pentaglycine and glycan chain, whereas CBD_A binds preferentially to pentaglycine cross-bridge, thus binds more selectively.

Binding domains of SpM23 enzymes are similar to well described and characterized prokaryotic sarcoma homology 3 binding domains (SH3b), which were found in Lss and ALE-1 hydrolases. SH3b were shown to have a well-defined binding groove that interacts selectively with staphylococcal PG cross-bridges (26). Upon comparing the surface of the CBD_B and CBD_A models and the CBD_Lss structure, we observed that CBD_B and CBD_Lss display similar sizes, wide binding grooves (80 and 76 Å^3^, respectively), whereas CBD_A binding groove has a rather limited volume (40 Å^3^, Fig. 5B). This could partially explain the wider binding spectrum of CBD_B and CBD_Lss and the narrow one of CBD_A.

Our competition assays demonstrate that CBD_A binds bacterial CW/PG less prominently than CBD_B or CBD_Lss because it inhibits the action of Lss only at the highest concentrations (Fig. 5A, Fig. S8). CBD_B seems not to bind to the very specific elements of the CW. Its interaction with the bacterial surface is presumably based on more universal features of the CW, such as for instance, the electrostatic interactions.

### CBD_B could extend the lytic activity spectrum of SpM23_A catalytic domain.

As demonstrated above, CBD_B bound the CW of a broad range of bacterial species, while CBD_A exhibited very narrow binding specificity (Fig. 4). Although the molecular basis of this difference is still not fully understood, we decided to test whether the CBD_B domain could be used as a universal anchor for other lytic domains. To test this hypothesis, we generated a chimeric enzyme made up of EAD_A and CBD_B (Fig. 5C).

The lytic activity of the generated chimera revealed that the CBD_B domain influenced the activity/specificity of EAD_A. The chimeric enzyme was less effective against those bacterial species, which were lysed efficiently by SpM23_A parental enzyme. At the same time, strains that were lysed by SpM23_B but not SpM23_A were susceptible to the chimeric enzyme (S. hominis, *S*. *cohnii*, *S*. *xylosus*, and *S*. *capitis*; Fig. 5C). Only *S*. *warnerii* was more effectively eliminated by the chimeric enzyme than SpM23_A and SpM23_B. Moreover, those strains that were not lysed by the parental enzyme SpM23 alone were also not susceptible to the chimera.

Overall, the chimeric enzyme specificity pattern was shifted from SpM23_A toward SpM23_B specificity, indicating that the CBD domain present in chimera controls lytic activity of the tested EAD.

### Lytic activity of the enzymes is tuned by the net charge of the bacterial cell surface.

**FIG 6:**
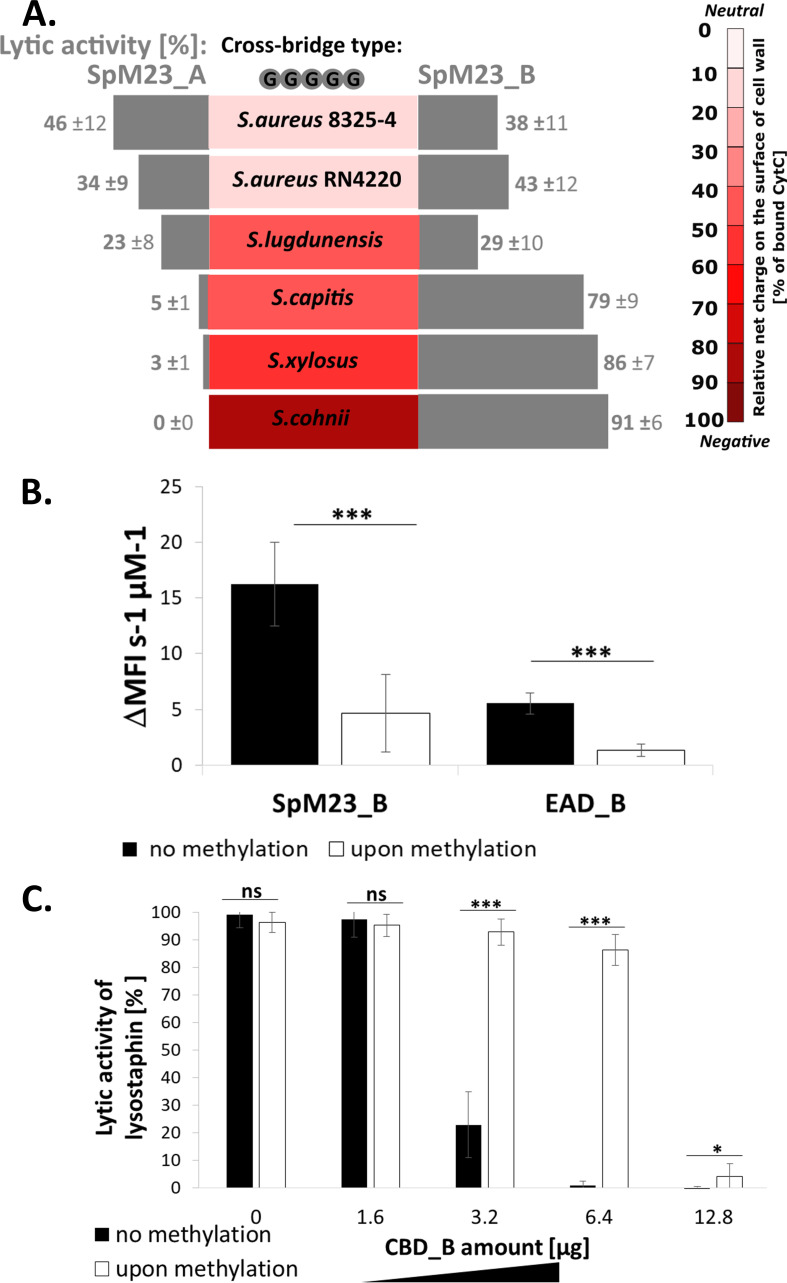
Role of charge in enzyme–CW interactions. (A) Comparison of enzymatic lytic activity and net charge on the bacterial cell surface measured in CytC assay. The lytic activity was presented as the reduction of initial OD_600_ after 1 h of incubation of bacteria in suspension with 100 nM enzyme in 50 mM glycine buffer pH 8.0, 100 mM NaCl and subtracted to negative control values (bacterial suspension incubated without added enzyme). The values of the measured net charge of the bacterial cell surface is presented as a spectrum of the percentage of the bound CytC fraction. The experiments were performed three times independently, and each bar represents the average ± standard deviation. (B) Comparison of lytic activity of SpM23_B and EAD_B before and upon methylation. SYTOX fluorescence assay performed in 50 mM glycine buffer with pH 8.0 and 100 mM NaCl at room temperature. The mean fluorescence intensity was calculated from the steepest linear region of the obtained lysis curve. The statistical significance of the differences was calculated using Student’s *t* test. ***, *P* < 0.01. (C) Comparison of the binding affinity of methylated and non-methylated form of CBD_B in the competitive assay with the Lss. The lytic activity was measured as the percentage of reduction of initial turbidity of S. aureus RN4220 cells incubated 1 h at room temperature with 100 nM Lss, preincubated with increasing concentration of CBD_B. The results were normalized to the negative control and incubated without added enzyme. The statistical significance of the differences was calculated using Student's *t* test. ***, *P* < 0.01; **, *P* < 0.1; ns, not significant.

Two bacteriolytic enzymes with a conserved amino acid sequence but distinct pI values create an excellent model for investigating the role of electrostatic interactions in the regulation of PG hydrolases activity. To analyze these phenomena, we tested SpM23 enzymes activity using a set of staphylococcal strains with diverse sensitivity to SpM23_A and SpM23_B but the same composition of cross-bridges (pentaglycine). We examined the surface net charge of those selected bacterial strains using CytC and performed activity assays using mature form of each enzyme, concluding that both EAD and CBD domains participate in overall bacteriolytic efficacy. A direct correlation between the charge of the bacterial surface and the activity of the enzymes was observed (Fig. 6A, Fig. S9). SpM23_A enzyme lytic activity was inhibited by decreasing negative charge of the bacterial surface (*r*(22) = 0.76, *P* < 0.000836). Likewise, the more negative a cell envelope is, the greater the observed activity of SpM23_B (*r*(22) = 0.55, *P* < 0.005672) (Fig. S9). These results demonstrate that the activity of the studied PG hydrolases is likely tuned by the net charge of the bacterial cell surface, which indicates that the electrostatic interactions between the enzyme and the bacterial CW play a role in hydrolase activity modulation.

Such interactions are determined by both the surface charge of the bacterial cell wall and the distribution of the charge on protein surface. To confirm our hypothesis, we have run a set of experiments in which the charge on the protein surface was altered by methylation while the charge on bacterial surface was modulated by modification of teichoic acids.

### Neutralization of the SpM23_B net charge diminishes its lytic activity.

We altered the pI of SpM23_B by methylating the lysine residues in the mature enzyme and its separate domains. Mass spectrometer analyses confirmed the desired modifications; all proteins subjected to this procedure had more than 90% of free amine groups di-methylated, which reduced their pI value by almost three units for each of studied enzymes (Table S2A, B). Because no lysine residues were found in the active center or in the binding groove, we assumed that the procedure did not affect the catalytic and binding efficiency of the enzymes. We confirmed that by FT-IR spectroscopy, which revealed no significant changes to the secondary structures of the methylated proteins (Table S2B, C).

The enzymatic activity was determined by SYTOX fluorescence assay on *S*. *capitis*, which was chosen as a substrate due to the prominent activity of the SpM23_B enzyme and the EAD domain against this strain. The lytic assay demonstrated that methylation significantly decreased the activity of the enzymes, both in their mature form and in the isolated catalytic domain (Fig. 6B). Methylation also had an impact on the binding of CBD_B, as measured in competition assay with Lss, analogously to that presented in Fig. 5A (Fig. 6C). Methylated CBD_B blocked the lytic activity of Lss less efficiently than the parental protein.

The neutralization of the charge upon methylation does not abolish lytic activity or binding affinity, but it does significantly diminish the optimal performance of both EAD and CBD. Overall, a positive charge enhanced the lytic activity and binding affinity of the SpM23_B enzyme.

### Teichoic acids affect binding and the lytic activity of the enzymes.

**FIG 7:**
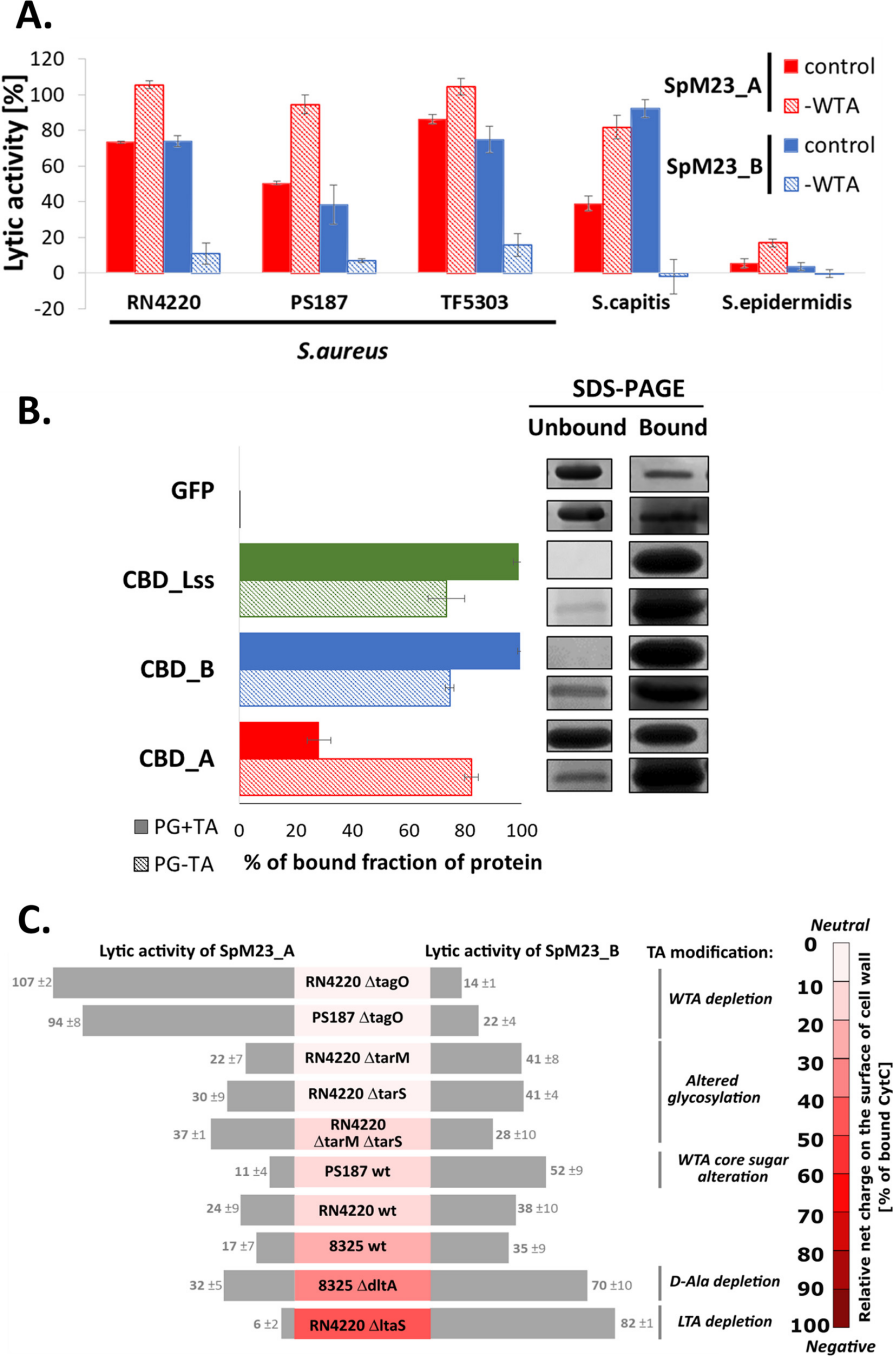
Teichoic acids affect binding and the lytic activity of the enzymes. (A) The effects of WTA depletion upon tunicamycin treatment on the lytic activity of SpM23_A and SpM23_B enzymes. The lytic activity is presented as percentage reduction of the initial turbidity after 1 h reaction incubation at room temperature (RT) in 50 mM glycine buffer pH 8.0. The negative control (incubation without added enzyme) was subtracted from the result presented. The experiments were performed three times and presented as mean values ± standard deviations. (B) Pull-down assay of CBDs and PG with or without TA. Left panel: Each CBD was mixed with the same volume of PG, with or without TA (removed upon trichloroacetic acid treatment), incubated for 1 h in PBS buffer at RT and then pelleted. GFP was used as the negative control. Abs280 was measured in the supernatant, and the percentage of protein bound to PG was calculated. Right panel: documentation of the SDS-PAGE gel presenting the bound (not diluted) and unbound (2.5× diluted) fractions. The run was performed on the 10% SDS-PAGE gel. (C) Lytic activity against a set of S. aureus strains with altered WTA or LTA synthesis pathways. The presented lytic activity results were obtained in a turbidity reduction assay after the incubation of 100 nM tested enzymes in 50 mM glycine buffer pH 8.0 with 100 mM NaCl at room temperature for 1 h. The surface net charge of the S. aureus teichoic acid mutants was estimated with CytC assay as described in Fig. 6A.

The main CW components involved in charge determination are anionic glycopolymers, namely, wall teichoic acids (WTA) and lipoteichoic acids (LTA). Hence, we have investigated how modifications of teichoic acid influence bacterial surface charge and weather this is reflected in changes in enzymes activity. Using CytC assay, we demonstrated that the charge on the bacterial surface is increased upon treatment with WTA synthesis inhibitor, tunicamycin, irrespective of the bacterial species (Fig. S10A). We have also compared the lytic activity pattern of SpM23 enzymes against wild-type and tunicamycin treated variants (Fig. 7A). WTA depletion for each bacterial strain tested resulted in the stimulation of SpM23_A activity, whereas the SpM23_B bacteriolytic activity was inhibited. The same was observed for the SpM23_B enzymatically active domain alone (Fig. S10B).

The lack of WTA in tunicamycin treated cells can be accompanied by alteration in CW architecture what may potentially affect enzyme activity (40). In order to exclude such effect, lytic assays were performed using bacterial cells of *S. aureus* RN4220 wt strain and its tagO mutant (ΔtagO) that does not synthesize WTA (41) additionally exposed to tunicamycin. We did not observe any prominent changes in the lytic activity of SpM23 enzymes between tunicamycin treated variants and ΔtagO mutant (Fig. S10C). It was also verified using PG as enzymes substrate, isolated from (i) S. aureus RN4220 cells with additional step of TA removal by trichloroacetic acid treatment, (ii) *S. aureus* RN4220 cells cultured in the presence of tunicamycin, and (iii) *S. aureus* RN4220 ΔtagO mutant cells (Fig. S10D). We obtained similar pattern of the enzymes activity and therefore concluded that the tunicamycin affects enzymes activity rather via modification of TA content than PG structure modifications.

Overall, we observed increased activity of the SpM23 enzymes against each PG variant with removed TA. The same effects were observed also on TA-depleted PG isolated from different staphylococcal strains (Fig. S10E). This suggests that TA present on PG block the activity of all tested enzymes, likely by impeding their access to the substrate. In contrast, TA present on the surface of the intact cell modulate the action of the enzymes in different way and different means, e.g., electrostatic interactions, which is observed as inhibition of SpM23_A activity but stimulation of SpM23_B.

These results indicate that variations in the charge of bacterial envelope strongly affect the activity of the enzymes and that WTAs are involved in this regulation. To verify whether this impacts binding efficiency as well, we performed a CBD competition assay with Lss on S. aureus RN4220 wt and ΔtagO mutant (Fig. S10F). The increasing amounts of CBD_A preincubated with bacteria blocked the activity of Lss, and this effect was enhanced in S. aureus lacking teichoic acid. The excess of CBD_B caused even stronger inhibition of Lss activity compared to the effect of CBD_A; however, the lack of teichoic acids reduced but did not completely abolish this effect. We supported this observation with the pull-down assay on the purified PG with or without TA, which showed that CBD_A binding was increased upon the WTA removal, while CBD_B and CBD_Lss was reduced (Fig. 7B). GFP alone does not follow the same rules what excludes the possibility of unspecific binding in the experimental conditions. However, it should be noted that upon removal of TA from PG the level of binding is similar for each tested CBD, what indicates that TA orchestrate their binding preferences.

Dramatic differences in the lytic activity and binding of the M23 enzymes upon the depletion of WTA prompted us to study the specific modifications of WTA that might affect interactions with the enzyme molecules. We analyzed the surface charge of TA synthesis pathway mutants using CytC assay and their susceptibility to SpM23_A and SpM23_B mature enzymes (Fig. 7C).

The wild type S. aureus strains (RN4220, 8325) display a negative surface charge reflected in the 16% of CytC binding. The most pronounced changes in charge among tested mutants was recorded for RN4220 ΔtagO, which displayed a diminished negative charge on the cell surface (6% of bound CytC), while surprisingly the mutant defective in LTA synthesis was the most negatively charged (RN4220 ΔltaS, 47% of bound CytC). These changes were clearly reflected in enzymes activity, the SpM23_A was the most active toward the RN4220 ΔtagO, whereas SpM23_B toward the RN4220 ΔltaS, which is in agreement with a previously demonstrated correlation (Fig. S9).

Knowing that the presence of TAs affect the SpM23_A and SpM23_B lytic activities, we wondered whether TA modification could have an impact on the enzyme activity, even though they do not alter the bacterial cell surface net charge substantially. S. aureus mutants altered in WTA glycosylation pattern (RN4220 ΔtarM, defective in α-glycosylation, and RN4220 ΔtarS, defective in β-glycosylation, RN4220 ΔtarMΔtarS, no glycosylation) were as susceptible to bacteriolytic activity of tested enzymes as the wild type S. aureus strains. The mutant that lacked the D-alanine moieties that decorate WTA was more prone to lysis by both PG hydrolases, with an apparently high activity of SpM23_B. The effects of altering the core sugar of WTAs (rybitol to glycerol) present in the PS187 strain of the unique S. aureus lineage ST395 did not significantly affect the hydrolases activity (Fig. 7C).

We examined the susceptibility of S. aureus to SpM23_A and SpM23_B during different growth phases (Fig. S11A) and observed very high SpM23_A activity at the lag- and log-phases (OD_600_ 0.2 to 0.6) while the highest activity of SpM23_B was recorded at log-phase (OD_600_ 0.6). Bacterial cells were less susceptible to both enzymes at stationary phase of growth, although to lesser extent to SpM23_A than to SpM23_B.

We probed the surface charge of the bacterial cells at certain growth phases and observed that indeed it is getting slightly neutralized in the course of culture aging (14% less CytC bound between lag and stationary phase; Fig. S11A). Similar changes were true also for other staphylococci tested (*S. aureus* RN4220 wild-type and, *S. capitis;* Fig. S11B). As the same effect were observed for *S. aureus* devoid of WTA (*S. aureus* RN4220 tagO mutant; Fig. S11B), we assumed that surface charge neutralization at stationary phase is WTA independent. It has been shown previously that the level of teichoic acids decreases while the thickness of PG increases when S. aureus enters stationary phase of growth (42).

To summarize, we observed that (i) the activity of both enzymes varies depending on the bacterial culture growth phase, (ii) SpM23_A and SpM23_B lyse preferentially bacteria at different stages of growth, and (iii) other factors than charge/TA content can also be involved in determination of susceptibility of bacterial culture at certain growth stage to SpM23 enzymes, e.g., increase of PG thickness.

## DISCUSSION

### Common and unique features of SpM23A and SpM23B enzymes.

SpM23 enzymes are PG hydrolases that disrupts Gly-Gly bonds within bacterial cross-bridges (Fig. 2). The specificity of SpM23A and SpM23B enzymes is limited to selected staphylococcal species, particularly in the case of SpM23_A, which can lyse only a very few of them. The very narrow lytic specificity of SpM23_A is a unique feature among M23 peptidases, which usually target broader spectrum of bacterial PG, like lysostaphin lysing most staphylococcal species or EnpA that target bacteria with diverse PG composition (15, 16). Interestingly, the CBD_B domain recognize and bind a much broader spectrum of bacterial species than SpM23_B lyse. In fact, CBD_B binds to all bacterial species tested, including even Gram-negative bacteria. In contrast, binding of CBD_A was limited predominantly to the species, which were also lysed by this enzyme. Similarly, the activity of other M23 enzymes has been shown to correspond directly to their binding specificity, as has been reported for Lss and ALE-1 (27, 43).

Detailed structural and biochemical studies have demonstrated that the specificity of CW binding domains from Lss and ALE-1 relies on the selective binding of the pentaglycine cross-bridges characteristic for Staphylococcus sp. (13, 26, 44–47). The very narrow specificity of the catalytic domains of M23 enzymes from *S*. *pettenkoferii* and the much broader binding specificity of their corresponding CBDs indicate that there must be some additional mechanisms involved in the process of the substrate recognition, either for catalysis or for binding.

### SpM23 proteins as a model for studying the role of electrostatic interactions between the enzymes and the bacterial CW.

**FIG 8:**
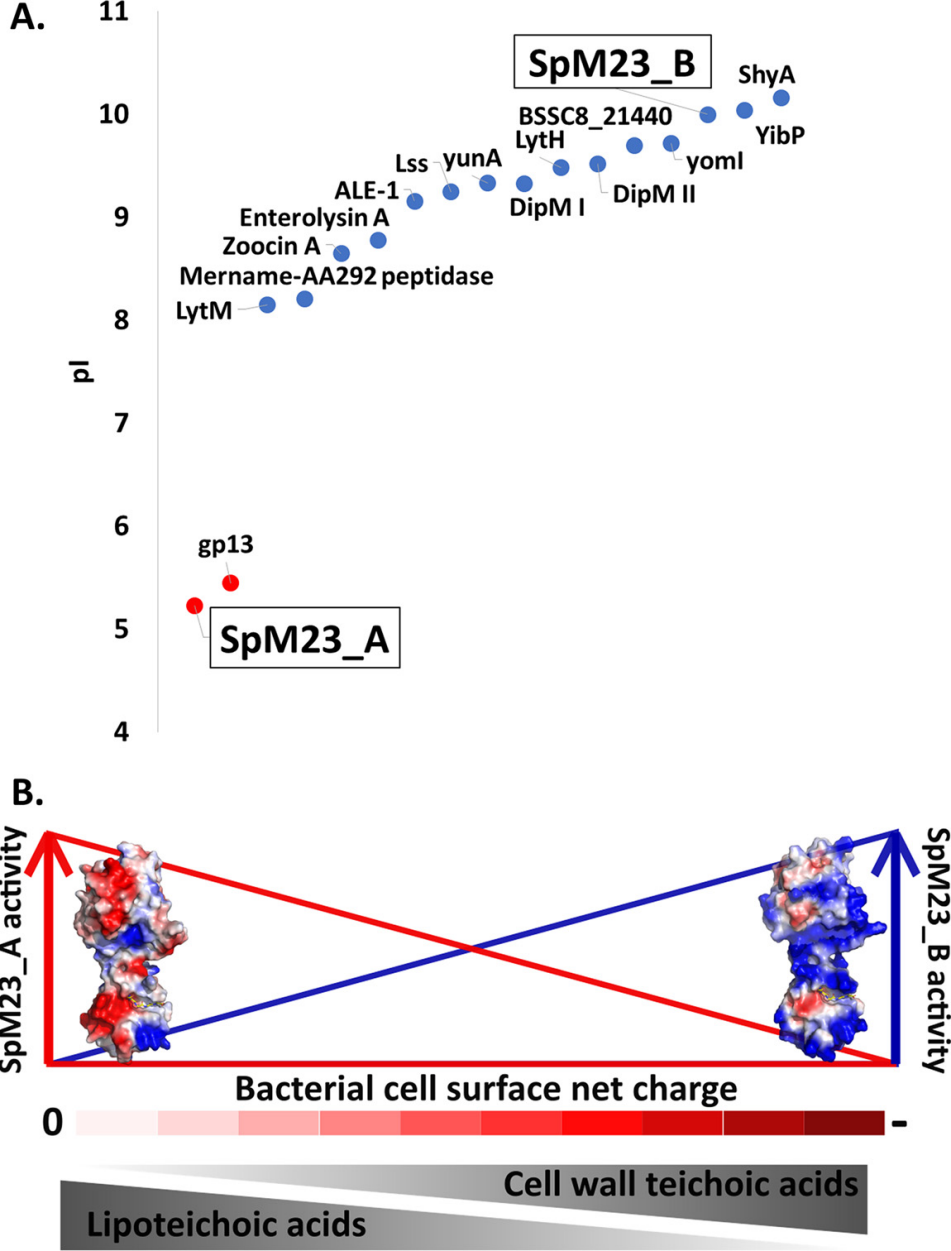
SpM23_A and SpM23_B are charged PG hydrolases, whose activity is modulated by the net charge on the bacterial surface and teichoic acids. (A) Distribution of the pI throughout the peptidase domains belonging to M23 family. The set of enzymes were selected according to the MEROPS database search (72), and their pI values were calculated in ProtParam (73) using the amino acid sequence of the peptidase domain identified by the MEROPS sequence identifier (MERNUM). The charge distribution was colour-coded, ranging from low (red) to high pI (blue). (B) Schematic representation of the factors impacting lytic activity of SpM23 enzymes, namely, electrostatic interactions with the bacterial cell surface and the presence of certain types of teichoic acids.

The vast majority of PG hydrolases described so far display a positive net surface charge, including peptidases from the M23 family (Fig. 8A). SpM23_A, which has a low pI value, is a very rare example among PG hydrolases. Apart from SpM23_A, the other member of the M23 family, the gp13 involved in the entry of bacteriophage phi 29 to the B. subtilis cell, is the only other example of low pI protein described so far (48).

The observation of positive charge on the surface of PG hydrolases and a negative one on bacterial CW has for many years been interpreted as a simple mechanism of providing electrostatic interactions that facilitate the attachment of the enzymes to the bacterial surface (4, 5). However, recently published results revealed that this relationship is not as simple and straightforward as was previously assumed (9, 49).

The relevance of the opposite charges of the surfaces of the CW and the PG hydrolases was elegantly demonstrated by Low et al. (5); using structure-based mutagenesis, the authors altered the protein surface charge and by that modulated the activity of the isolated catalytic domains of endolysins. Only proteins carrying a positive charge displayed bacteriolytic activity. In addition, enhanced lytic activity was reported after increasing positive surface net charge of the CW binding module of the Cpl-7 phage lysozyme (4). However, an increase in the net surface charge of the PlyC catalytic domain (CHAP) from −3 to +7 did not improve the performance of the enzyme, leading to the conclusion that the surface charge cannot be considered the only mechanism that regulates the interactions between the enzyme and the bacterial CW (9). The change in charge on the surface of both domains of Lss indicated much more complex relationships between charge effect, lysin electrostatics, bacterial targeting, and lysis efficiency (49).

The reports discussed above were based on enzymes on which the surface charge was genetically engineered, which could be accompanied by unintended changes affecting the results. To avoid these complications, we have used two enzymes that we have recently characterized as a model because they share multidomain architecture and high amino acid sequence homology, while at the same time, they display very different isoelectric points. This constitutes a good, natural model for studying the relevance of the enzymes’ surface charge to their interactions with bacterial CW.

Our studies brought us to the conclusion that the charge of bacterial envelope affect the lytic performance of the peptidoglycan hydrolases and thus define the spectrum of susceptible bacterial species (Fig. 6A, Fig. S9). This conclusion is supported by several independent observations revealing a strong relationship between the surface charge of cell and proteins as (i) tested on the whole spectrum of differently charged strains (Fig. 6A), (ii) manipulating protein charge by methylation (Fig. 6B and C), and finally (iii) modulating charge on the bacterial surface by changes in TA composition (Fig. 7C, Fig. S10A).

The unique point about these two proteins is the fact that their genes are present in the genome of the same bacterial strain, *S*. *pettenkoferi* VCU 012. This raises the question of the potential physiological roles of these two proteins, which we have addressed previously (10). The analysis on the genetic context of the genes encoding both enzymes, their distribution among *S. pettenkoferi* strains and *in vivo* activity of the enzymes suggest different roles for these two enzymes (10). We have proposed a role of bacteriocine for SpM23_B, which has broader specificity and narrower distribution compared with SpM23_A and, therefore, could be considered a better weapon in the battle with closely related species. By contrast, the SpM23_A, which displays much more limited activity but is present in all genomes of *S. pettenkoferi* strains, could be regarded as a precision instrument in CW remodeling. If this understanding were correct, charge modulation would be one of the ways to differentiate the roles assigned to each enzyme. In support for this, the lytic activity in the pH spectrum (Fig. S6) indicates that both enzymes are active in similar pH conditions. Thus, the differences in net charge can be used to harness their lytic activity rather than to tune their lytic activity to the particular environmental conditions.

### Complexity and diversity of the bacterial CW charge.

The positive charge on the surface of PG hydrolases is thought to provide a better affinity toward its target, namely, the bacterial cell envelopes, which are negatively charged due to the presence of surface carbohydrates (50–52). Thanks to the diverse modifications of the CW elements, such as the amidation of free carboxylate groups on the PG (53) or teichoic acid D-alanylation (54), the net charge on the bacterial surface can vary between species and strains. Moreover, this feature can also change during bacterial cell growth and has been shown to depend on environmental conditions (6, 55). In fact, we have described a wide range of cell surface negativity among the bacterial species and strains used in our studies, as measured using CytC assay (Fig. 6A, 7C).

The interactions between PG hydrolases and their ligands take place in the complex environment of the bacterial CW. Highly abundant teichoic acids, which may constitute even 60% of CW mass (2), are involved in CW surface net charge homeostasis (56, 57). Taking profit from several genetic mutants reported in recent years (32, 58) and by chemically inhibition of TA synthesis with tunicamycin, we could observe that WTA and LTA depletion had a very strong effect on the surface charge and as a consequence on the activity of both SpM23_A and SpM23_B.

As reported before, lack of TA may lead to pleiotropic effects and affect the cell surface properties, like level of peptidoglycan cross-linking (6, 57–60) or divalent ion homeostasis (61) that raises the question whether it would affect performance of the enzymes. Nevertheless, because both enzymes target the same structure in PG (10) (Fig. 2) and the effect exerted on them by each TA mutation is opposite, we rationalized that the impact of TA and its modifications is directly linked to alteration in bacterial surface charge, rather than indirectly via changing other cell wall properties. We confirmed that by i.e., probing activity of the enzymes against purified PG from the mutants in TA biosynthesis pathway (Fig. S10G) and observed that they are similarly active, meaning that possible alteration in PG composition does not affect the performance of the SpM23 enzymes.

The role of TAs in the regulation of PG hydrolases activity is a well described phenomenon (6, 28, 62, 63). As postulated, TA control autolysins activity by limiting it to specific time (64) and/or space (28, 62). This precise regulation of autolysins is extremely important for avoiding the uncontrolled disruption of the cell envelope integrity (65). Different molecular mechanisms of interactions between TA and enzymes have been proposed, including enzyme extracellular translocation control (66) or direct effects on lytic activity. Electrostatic interactions (28, 30, 67), mechanical restriction of the access to the PG (68) or stereochemical differences in TA (69) have all been proposed to affect the performance of PG hydrolysis. Our results contributed to the discussion of the charge-based regulation of enzyme activity by TA. This hypothesis assumes that TA acts as cation buffers (70) and modulate local pH (67, 71). As a consequence, a specific, local environment is established to control the performance of CW-associated proteins.

Indeed, upon WTA or LTA deletion (Fig. 7C), the bacterial surface charge was massively altered; while WTA depletion increased the surface charge, the inhibition of LTA synthesis resulted in its decrease. Similar modification of bacterial surface charge due to WTA/LTA depletion were observed in susceptibility of S. aureus mutants to Congo red and other anionic azo dyes (74). The possible explanation can come from the fact that although LTA and WTA are both considered to have an anionic nature, at the same time they might be differentially modified. Indeed, nuclear magnetic resonance showed that S. aureus LTA are ~85% D-alanylated (75), and WTA are much less decorated with D-Ala than LTA (30). Results of the CytC assay and the activity pattern of both SpM23_A and SpM23_B against LTA deletion mutant were similar to those observed for the mutant with blocked D-alanylation pathway (Fig. 7C). Changes in the bacterial surface charge were clearly reflected in SpM23_A and SpM23_B activity, once again demonstrating a direct link between the surface charges of the enzymes and the bacterial cell walls.

The strong LTA and WTA dependence of SpM23 enzymes resemble the phenomena described by Flores-Kim et al. (64) for LytA, a major autolysin of Streptococcus pneumoniae. They showed that LTA predominance appears in the early stages of bacterial growth and as it progresses, the equilibrium shifts to WTA predominance. Combining observations from both the activity assays against genetic mutants (Fig. 7C) and the ones performed on the bacteria at different growth phases (Fig. S11), we hypothesize that SpM23_A and SpM23_B lytic activity may be affected by changing proportions of the LTA and WTA during cell culture growth. The mild neutralization of bacterial surface charge does not follow LTA-WTA equilibrium found in *S. pneumoniae*, but it is in agreement with reported decrease of overall TA content of *S. aureus* culture at stationary phase of growth (42). Thus, although both SpM23 enzymes display varied, and different from each other, susceptibility toward bacteria at different growth phases, the underlying reason of that remains an open question.

### Conclusions.

SpM23_A and SpM23_B are a perfect model for verifying the previously postulated importance of the net charge for the regulation of PG hydrolase activity. By modifying the charge on either the bacterial or enzyme surface, we were able to demonstrate the importance of the electrostatic interactions involved in enzyme anchoring and lysis performance. Taking into account that both *spm23* genes are localized in the same bacterial genome and that the enzymes display dependence on the chemical proprieties of teichoic acids (Fig. 8B), electrostatic interactions would be the means of tuning the lytic activity of SpM23 enzymes in the bacterial CW context. Our results shed light on this aspect of the PG hydrolase regulation, which is dominated in the literature by artificially engineered enzymes.

The unique properties of SpM23 enzymes make them attractive compounds with huge application potential. SpM23_A displays a potent lytic activity spectrum limited to S. aureus, leaving unharnessed the natural microflora and beneficial commensal species, e.g., S. epidermidis. However, CBD_B also serves as a perfect platform for developing the antimicrobials of extended specificities, as demonstrated in the chimera example in this study. Taking its broad binding spectrum into account, it could likely act effectively in concert with catalytic domains displaying specificities that are even broader than the pentaglycine cross-bridge.

## MATERIALS AND METHODS

### Bacterial strains and growth conditions.

All bacterial strains were purchased from Polish Collection of Microorganisms, Institute of Immunology and Experimental Therapy, Wrocław, Poland; the German Collection of Microorganisms and Cell Cultures; the American Type Culture Collection; and the Czech Collection of Microorganisms, Masaryk University, Brno, Czech Republic. Others were kindly provided by the institutions listed (Table S1A).

E. coli was cultured in lysogeny-broth (LB) medium at 37°C with 250 rpm agitation. All other strains were cultured in tryptic soy broth (TSB) medium at 37°C with 80 rpm agitation.

### Molecular biology.

The DNA encoding for SpM23_A and SpM23_B was amplified, as described earlier (10). The chimeric enzyme was obtained using overlap extension PCR (20) and cloned into pMCSG7 vector (21), compatible with ligation-independent cloning. The EAD_B was amplified using the *spm23_B* as the template and cloned into pMCSG7 vector. To obtain the GFP fusion protein on the C-terminus, each of the CW binding domains (CBD) was cloned in XhoI-BamHI sites with expression of pWALDO vector (22) and confirmed by sequencing. All genetic constructs used in this work are listed in Table S1B.

### Protein expression and purification.

E. coli BL21(DE3) was transformed and plated on LB-Agar upon an antibiotic selection, and the single colony was inoculated in the overnight culture in LB medium with 250 rpm agitation at 37°C. The following day, the inoculum was refreshed, and the large culture grew under the same conditions until it reached an OD_600_ of 0.6. Upon induction with 1 mM isopropyl β-D-1-thiogalactopyranoside (IPTG), the temperature was decreased to 18°C, and the protein production was continued overnight. The bacterial pellet with overexpressed proteins was suspended in 20 mM Tris-HCl pH 7.0 (for CBD_B pH 9.0) and 1 M NaCl (buffer A), and the cells were sonicated. The crude lysate was clarified by centrifugation at 95,834 × *g* and the supernatant containing the proteins SpM23_A, SpM23_B, CBD_A, or CBD_B was dialyzed overnight to 20 mM Tris-HCl pH 7.0, 80 mM NaCl (buffer B). Next day, the dialyzed lysate was filtered and loaded onto the WB40S (Bio-Works, Sweden) or DEAE resin (Cytiva, USA) and the fractions corresponding to the peaks were collected upon gradient to the buffer A. The lysate of chimeric enzymes and EAD_B were loaded on the HisTrap columns (Ni Sepharose High Performance HisTrap HP, GE Healthcare, USA) and the gradient to 300 mM imidazole in 20 column-volume (CV) was run. The digestion with tobacco etch virus protease was performed overnight at 4°C upon dialysis to 20 mM Tris-HCl pH 7.0, 200 mM NaCl, 15 mM imidazole. The next day, the dialyzed samples were loaded on the HisTrap HP column and the flow-through was collected for further analyses.

After first purification step, all of the proteins were subjected to size exclusion chromatography in 20 mM Tris-HCl pH 7.0 (for CBD_B pH 9.0), 200 mM NaCl, 10% glycerol on a Superdex75 (GE Healthcare, USA) column, using an ÄKTA^TM^ Purifier system (Cytiva, USA). The purity of recombinant enzyme was assessed on the SDS-PAGE 10% gel and imaged with Coomassie-blue staining. The peak fractions were concentrated in Amicon centrifugal filters (Merck, Germany) and used for activity tests. For long-term storage, the proteins were flash-frozen in liquid nitrogen and kept at −80°C.

### CW and PG purification.

The protocol was established based on previously published CW components purification protocols (23, 24). TSB medium was inoculated with single bacterial colony and left overnight at 37°C with 100 rpm agitation. The cultures were centrifuged and the cell pellet washed three times with buffer C (20 mM ammonium acetate, pH 4.6). The wet weight of the obtained pellet was determined, and 4.5 g glass beads (Sigma) and 2 mL buffer C were added per 1 g of cells. The samples were aliquoted to 1.5 mL Eppendorf tubes and vigorously mixed in the Disruptor Genie for 30 min (Scientific Industries, USA). The obtained lysate was briefly centrifuged, the soluble fraction between foam and glass beads was collected and supplemented with MgSO_4_ (2 mM) and 750 U viscolase (A&A Biotechnology, Poland). The samples were left overnight at 37°C with 600 rpm agitation. The next day, SDS (2% final) was added to the sample and incubated for 1 h with 600 rpm agitation. After that, it was centrifuged and washed 10 times with buffer C. In order to remove teichoic acids, trichloroacetic acid (5% final) was added to the samples and incubated for 4 h a 65°C. After that the samples were washed with water until pH became neutral. The OD_600_ of the samples was measured.

### Lytic and binding assays. (i) Turbidity reduction assay.

A single colony of each bacterial strain was inoculated in small-volume overnight culture in TSB and cultivated at 37°C at 80 rpm agitation. The next day it was refreshed in a new portion of TSB, and the bacteria were cultured to the mid-exponential phase of growth (OD_600_ 0.6 to 0.8). For the assays performed at different phases of bacterial growth, cultures were collected at various time points. For the tunicamycin-treated variants, tunicamycin (Sigma# T7765, Merck, Germany) stock stored at −20°C was added to the final 0.2 μg/mL concentration at the onset of the bacteria culture. For the assays at different pH values, a set of buffers of the same conductivity (0.5 mS/cm) was prepared as follows: 10 mM acetate buffer pH 5.0, 2 mM citrate buffer pH 6.0, 5 mM Tris-HCl pH 7.0, 10 mM Tris-HCl pH 8.0, 50 mM glycine buffer pH 8.0, and 50 mM Tris-HCl pH 9.0.

To stop bacterial growth, the culture was pelleted and resuspended in a lytic buffer (50 mM glycine-NaOH pH 8.0). In high-ionic-strength conditions, the buffer was supplemented with 100 mM NaCl. The culture cell density was adjusted to around 10^8^ CFU/mL (OD_600_ ~ 1). The enzymes were added to the final concentration of 100 nM, and turbidity reduction was monitored every 10 min at 620 nm at a TECAN Infinite F50 microplate-reader (TECAN, Switzerland). The test was performed on a 96-well plate at room temperature. The results were compared with the negative control sample with the addition of the same volume of lytic buffer without the enzyme. All of the experiments were performed at least three times in triplicate.

**(ii) SYTOX fluorescence assay.** The bacterial culture was prepared as described above. The reaction was performed as described in Jagielska et al. (25). Briefly, bacterial pellet was resuspended in 50 mM glycine-NaOH pH 8.0, 100 mM NaCl, 1 μM SYTOX Green (Invitrogen, USA) and 50 nM or 100 nM each enzyme. The fluorescence was measured at room temperature for 1 h in 96-well plate reader (excitation: 504 nm, emission: 523 nm, Infinite M1000, TECAN, USA). The steepest linear region of the lysis curve served for the mean fluorescence intensity (MFI) calculation. The analyses were performed tree times.

**(iii) Competition with lysostaphin or mutanolysin.** The assay was adopted from Mitkowski et al. (26). Bacterial cultures of S. aureus (Lss assay) and Streptococcus agalactiae (mutanolysin assay) were prepared as described above and resuspended in PBS (pH 7.2) and the turbidity of the culture was adjusted to OD_600_ 1 (around 10^8^ CFU/mL). Bacterial cell suspension was mixed with increasing amounts of each CBD and left at room temperature for 15 min. Then, 100 μL tested mixture was transferred to a 96-well plate. The Lss or mutanolysin (A&A Biotechnology, Poland) solutions were added to the mixtures in a 1:1 (vol/vol) ratio at final concentration of 100 nM. The lytic activity was compared to the negative control (bacteria incubated with the highest concentration of CBD but without an added enzyme) and the positive control (bacteria not pre-incubated with CBD but treated with bacteriolytic enzyme). All of the experiments were performed three times with at least two replicates.

**(iv) Binding assay.** The assay was performed using the protocol from (27). Briefly, the bacterial cell cultures were prepared as described above and resuspended in PBS (pH 7.2). The cell suspension turbidity was adjusted to OD_600_ 20. Then, 130 μL bacterial cell suspension was mixed with each CBD_GFP domain (in a 1 μM final concentration), incubated at room temperature for 15 min, and then the bacteria were pelleted. The negative control samples, namely, A, bacteria without added enzyme, and B, each CBD_GFP domain in 150 μL the PBS buffer alone, were also included. The supernatant was treated as an unbound fraction, and the fluorescence intensity was measured in the 96-well plate reader (excitation, 488 nm; emission, 508 nm; Infinite M1000, TECAN). The obtained values were normalized to negative control B, and the percentage of the bound protein fraction was calculated. The emission of the negative control A was also included in those calculations. All of the experiments were performed at least three times in triplicate.

**(v) Imaging with confocal microscopy.** The assay was based on the protocol from (27) and (28). Briefly, the bacterial cell cultures were prepared as described above and resuspended in PBS (pH 7.2). The cell suspension turbidity was adjusted to OD_600_ 1. Then, 20 μL of sample containing selected GFP-fused binding domain (250 nM), bovine serum albumin (BSA, 10 mg/mL), and PBS were mixed with 80 μL of washed S. aureus or E. coli. The samples were incubated for 10 min at room temperature. Bacteria and the bound protein were sedimented by centrifugation at 16,000 × *g* for 3 min. The supernatant was discarded and the pellet was resuspended in 100 μL PBS. The cells were plated on microscope glass (10 μL) and sealed with nail polish immediately before microscope observation. The images were obtained using LSM 800 (Zeiss, Germany) using a 63× objective and Airyscan detector.

**(vi) Pull-down assay.** The assay was performed using PG extracted from S. aureus TF5303 with retained or removed teichoic acids. The variant without teichoic acids was subjected to trichloroacetic acid treatment. From each CBD sample, 10 μg was mixed with 5 μL PG sample OD_600_ ~ 20, and the reaction was topped up to 50 μL with PBS buffer, pH 7.2. The samples were left at room temperature for 1 h with mixing every 20 min. After that, the samples were centrifuged and the absorbance at 280 nm was determined for the unbound fraction. Both unbound (20 μL of the 50 μL sample) and bound (all) fractions were run on the 10% SDS-PAGE gel and imaged as described above.

**(vii) Remazol brilliant blue R dye release assay.** The assay was based on the method described by Zhou et al. (29). Briefly, Remazol Brilliant Blue R (RBB, Sigma# R8001, Merck, Germany) was dissolved in 250 mM NaOH to a final concentration of 20 mM. PG sample was incubated with the staining mixture for 6 h at 37°C and then around 12 h at 4°C. PG sample was thoroughly washed with water until supernatant became transparent. The lytic assay on Remazol-dyed PG was performed for 1 h or overnight at 37°C. Amount of PG was adjusted to OD ~6. For the competition assays, the PG samples were weighted prior to the reaction, adjusted to 180 μg per sample and then incubated for 5 min with CBDs. PG samples were mixed with the same volume of the enzyme (100 nM final concentration for SpM23 enzymes and Lss and 100U for mutanolysin). Reaction was quenched with equal volume of 96% ethanol, sample was centrifuged and the absorbance of the supernatant was measured at 595 nm.

**(viii) Pentaglycine and muropeptides digestion assays.** Pentaglycine (Sigma #G5755, Merck, Germany) solution was solubilized in 50 mM glycine pH 8.0 buffer upon heating the sample in 95°C for a minute. Final concentration of 2 mM pentaglycine was mixed with 4 μM enzymes or buffer (control sample) in 50 mM glycine pH 8.0 buffer, 100 mM NaCl. The samples were left overnight in 37°C. Next day, samples were subjected to mass spectrometry (MS) analysis. Results were analyzed in MassLynx (v 4.1).

Muropeptides were generated upon overnight digestion of PG purified from *S. aureus* NCTC 8325-4 in 50 mM glycine pH 8.0 buffer, 100 mM NaCl. Equal volume of solubilized PG was mixed with 4 μM enzymes and incubated for 24 h at 37°C. Prior to high-performance liquid chromatography (HPLC)-MS, samples containing muropeptides were reduced. Results were analyzed in MassLynx (v 4.1).

### Cytochrome c assay.

The assay was performed based on the protocol from (30). Briefly, a single colony of each bacterial strain was inoculated in 20 mL TSB and cultivated at 37°C with 100 rpm agitation. On the following day, the culture was pelleted and resuspended in reaction buffer, 20 mM ammonium acetate buffer pH 4.6. The cell density was adjusted to the same value (OD_600_ ~ 30), and 600 μL of bacterial suspensions were pelleted. The pellets were resuspended in 0.2 mg/mL cytochrome c (CytC) solution in reaction buffer and incubated for 15 min at 37°C. Next, the sample was pelleted, and the supernatant immediately measured at 410 nm using Infinite®F50 microplate-reader (TECAN, Switzerland). The measurement was performed on 96-well plate at room temperature. The results were compared with the CytC sample without added bacteria, which was the negative control. All experiments were performed at least three times in triplicate.

### Methylation of the enzyme.

The methylation of the enzyme was performed by dialyzing the protein sample against PBS, incubating it with dimethylamine borane complex (DMAB) solution and formaldehyde, as described (31). For the untreated sample, an equal volume of PBS was added instead of DMAB and formaldehyde. After the methylation was complete, both samples were dialyzed against PBS and concentrated in Amicon centrifugal filters. The Fourier-transform infrared spectroscopy was performed in an FT-IR Spectrometer (TENSOR Series, Bruker, USA).

### Data availability.

All data generated or analyzed during this study are included in this published article and its supplementary information files. The data sets generated during and/or analyzed during the current study are available from the corresponding author on reasonable request.
