# Supplementary material for: Electrostatic Interaction with the Bacterial Cell Envelope Tunes the Lytic Activity of Two Novel Peptidoglycan Hydrolases
Source: Microbiol Spectr. 2022 Apr 25;10(3):e00455-22. doi: 10.1128/spectrum.00455-22 (PMC9241647; doi:10.1128/spectrum.00455-22)
Supplement: SUPPLEMENTAL FILE 1 — Supplemental material. Download spectrum.00455-22-s001.pdf, PDF file, 2.2 MB [file spectrum.00455-22-s001.pdf]

Supplementary Information

Table S1. The list of materials used in this study. The information on the cross-bridge composition was collected from database of Leibniz Institute DSMZ-German Collection of Microorganisms and Cell Cultures GmbH.

A. Bacterial strains.

Species	Strain reference number/isolate/mutation	Cross-bridge composition	Reference
Escherichia coli	TOP10	Direct cross-link	-
Escherichia coli	BL21 (DE3)	Direct cross-link	-
Bacillus subtilis	ATCC 6633	Direct cross-link	-
Corynebacterium striatum	DSM 20668	Direct cross-link	-
Lactococcus lactis	CCM 1877	D-Asp	-
Micrococcus luteus	ATCC 10240	Direct cross-link (L-Ala-D-Glu(Gly)-L-Lys-D-Ala)	-
Propionibacterium (Cutibacterium) acnes	PCM 2334	LL-Dpm-Gly	-
Streptococcus agalactiae	123B	L-Ala_{2-3}	-
Acinetobacter haemolyticus	PCM 2113	Direct cross-link	-
Staphylococcus aureus	NCTC 8325-4	L-Lys-Gly_{5-6}	-
Staphylococcus aureus	NCTC 8325 and ΔdltA mutant*	L-Lys-Gly_{5-6}	(1)
Staphylococcus aureus	TF 5303<br>TF 5311	L-Lys-Gly_{5-6}<br>L-Lys-Gly_2-L-Ser_1-Gly_2	(2)
Staphylococcus aureus	8325<br>8325 BB815 Δ*femB*<br>8325 AS145 Δ*femAB*	L-Lys-Gly_{5-6}<br>L-Lys-Gly_3<br>L-Lys-Gly	(3)
*Staphylococcus aureus**	RN4220 wt and mutants :<br>• ΔtagO<br>• ΔtarM<br>• ΔtarS<br>• ΔtarMΔtarS<br>• ΔltaS	L-Lys-Gly_{5-6}	(4)<br>(5)<br>(6)
*Staphylococcus aureus**	PS187 wt and ΔtagO mutant	L-Lys-Gly_{5-6}	(7)
Staphylococcus argensis	DSM 29875	L-Lys-Gly_{5-6}#	
Staphylococcus capitis	PCM 2121	L-Lys-Gly_{5-6}#	-
Staphylococcus cohnii	DSM 20260	L-Lys-Gly_{5-6}#	-
Staphylococcus epidermidis	DSM 20044	L-Lys-Gly_{2-4}-L-Ser_{1-2}-Gly#	-
Staphylococcus epidermidis	RP-62A	?	(8)
Staphylococcus haemolyticus	PCM 2113	L-Lys-Gly_{5-6}#	-
Staphylococcus hominis	PCM 2122	L-Lys-Gly_{5-6}#	-
Staphylococcus intermedius	DSM 20373	L-Lys-Gly_{5-6}#	-
Staphylococcus lugdunensis	PCM 2430	L-Lys-Gly_{5-6}#	-
Staphylococcus pasteuri	PCM 2445	L-Lys-Gly_{2-4}-L-Ser_{1-2}-Gly#	-
Staphylococcus pettenkoferi	DSM 19554	?	-
*Staphylococcus pettenkoferi***	VCU012	L-Lys-Gly_{2-4}-L-Ser_{1-2}-Gly#	
*Staphylococcus pettenkoferi****	Vasteras_17:2, 17:5, 17:8, 18:1	?	(9)
Staphylococcus saprophyticus	PCM 2109	L-Lys-Gly_{5-6}#	-
Staphylococcus simulans	CCM 3583	L-Lys-Gly_{2-4}-L-Ser_{1-2}-Gly#	-

Staphylococcus simulans	DSM 20037	L-Lys-Gly_{5-6}#	-
Staphylococcus warneri	PCM 2107	L-Lys-Gly_{5-6}#	-
Staphylococcus xylosus	PCM 2114	L-Lys-Gly_{5-6}#	-

Abbreviations: Dpm - Dpm, 2,6-diaminopimelic acid
* kind gift of the Department of Infection Biology, University of Tübingen
** kind gift of the University of Virginia, USA; the cross-bridge analysis was based on unpublished Liquid chromatography–mass spectrometry analysis (*not published data*)
*** environment isolates, kind gift from Department of Orthopedics, Central Hospital Vasteras, Sweden
# The cross-bridge composition for this staphylococci strain was deduced based on the lysostaphin susceptibility assay (Figure S3).

B. Constructs and proteins.

Plasmid	Produced protein variant, abbreviation	Fused tag	Region, *length* [aa]	Theoretical pI	Molecular weight [Da]
pET22b	Mature form, SpM23_A	-	217-457, *240*	5.68	26455.96
pET22b	Cell wall targeting domain, CBD_A	-	365-457, *93*	6.10	10524.68
pWALDO	Cell wall targeting domain fused to GFP, CBD_A_GFP	C-terminal GFP and his-tag	365-457, *351*	6.38	39601.30
pET22b	Mature form, SpM23_B	-	199-446, *248*	10.25	27194.00
pET22b	Cell wall targeting domain, CBD_B	-	354-446, *93*	10.36	10540.22
pWALDO	Cell wall targeting domain fused to GFP, CBD_B_GFP	C-terminal GFP and his-tag	354-446, *351*	9.17	39675.91
pET22b	Cell wall targeting domain, EAD_B	-	217-348, *131*	5.23	14460.85
pET22b	Mature form, Lss	-	209-452, *245*	9.59	26700.98
pET15b	Cell wall targeting domain, CBD_Lss	-	361-452, *93*	9.85	10369.72
pMCSG7	Acidic enzymatically active domain, EAD_A and Basic cell wall targeting domain with linker, CBD_B; Chimera	N-terminal his-tag, TEV protease cleavage site	EAD_A: 217-348, *131* Linker+BD_B: 337-446, *109*	9.78	26183.37

Table S2. Assessment of the protein characteristics before and after methylation.

A. Mass spectroscopy (MS) measurement of the methylated proteins. The theoretical pI was calculated using the IPC2 (Isoelectric Point Calculator 2.0) (10) and marked in red. For the calculation of methylated proteins pI all lysins were converted into glycines. The number of presented residues concern only lysins (the N-terminal amine group methylated in this procedure was excluded from the analysis).

B. Comparison of MS graphs indicating shifts in the protein sample mass in control (- methylation) and methylated variants (+methylation). Fourier Transform Infrared (FTIR) Spectroscopy graphs comparing the secondary structure composition of the protein samples not subjected (blue) and subjected (red) to chemical methylation procedure are in right panel. The number of demethylated lysin residues (marked with star *) against total number of free lysin residues, and percent of lysin residues methylated (%) are indicated for the identified peaks, the methylation of N-terminal amine group was excluded.

C. FTIR result table showing percent composition of secondary structures in protein sample not subjected (**-**) and subjected (**+**) to chemical methylation procedure.

A.

	**Molecular weight before methylation [Da]/pI**		**Molecular weight after methylation [Da]/pI**		**Number of demethylated lysin residues /total number of free lysin residues (%)**
SpM23_B	27320.50	**9.44**	28105.00	**6.57**	*27/30 (90)
EAD_B	15067.00	**9.43**	15431.00	**7.33**	*12/12 (100)
CBD_B	10612.50	**10.97**	11033.00	**8.14**	*14/14 (100)

**B.**	**Mass Spectrometry chromatograms**		**Fourier Transform Infrared (FTIR) Spectroscopy**
Protein	**- methylation**	**+ methylation**	
SpM23_B	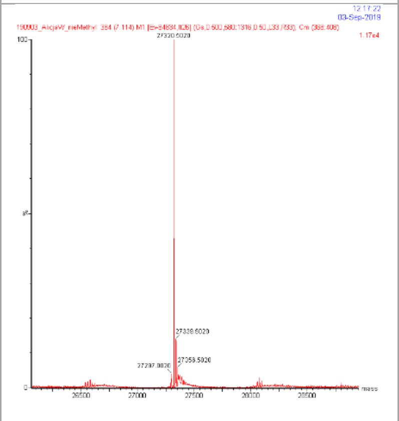	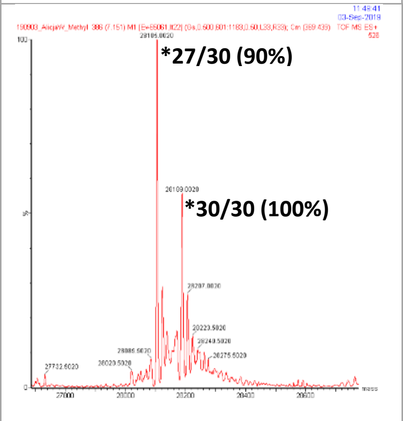	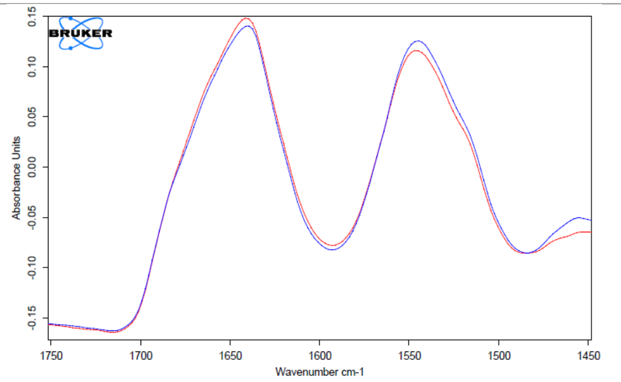
EAD_B	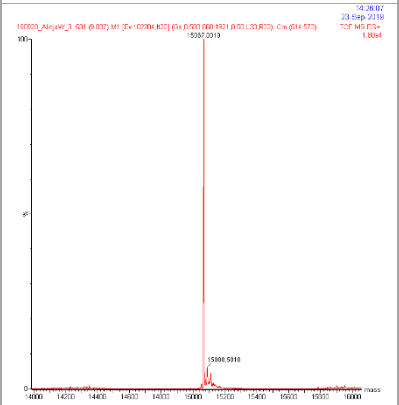	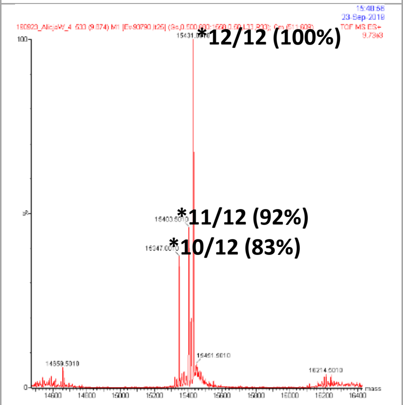	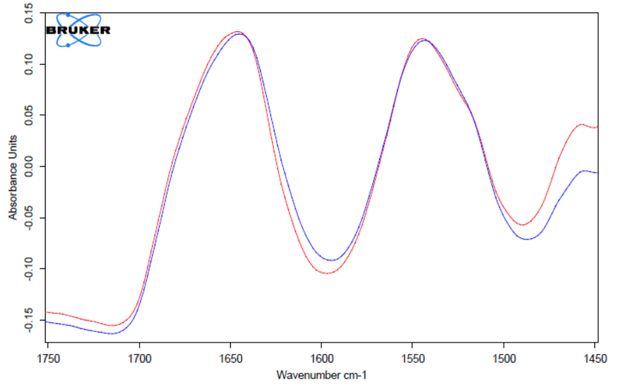

CBD_B

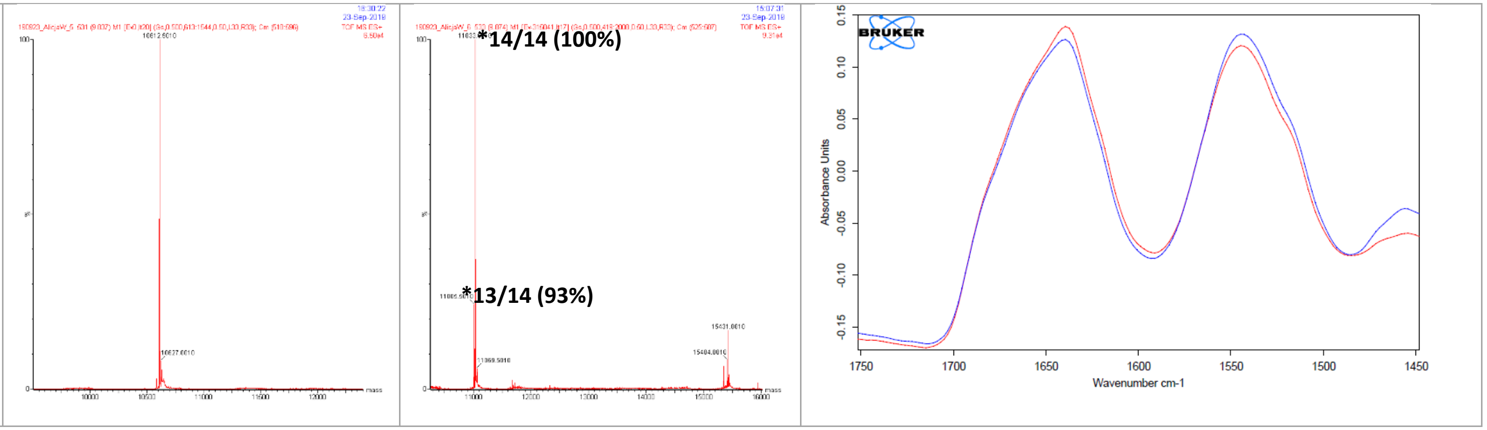

C.

Protein	Component	Methylation	Prediction [%]
SpM23_B	alpha-helix	-	6.3
		+	6.2
	beta-sheet	-	34.5
		+	34.5
EAD_B	alpha-helix	-	0.2
		+	3.0
	beta-sheet	-	27.1
		+	22.4
CBD_B	alpha-helix	-	3.3
		+	1.8
	beta-sheet	-	35.3
		+	36.9

A.

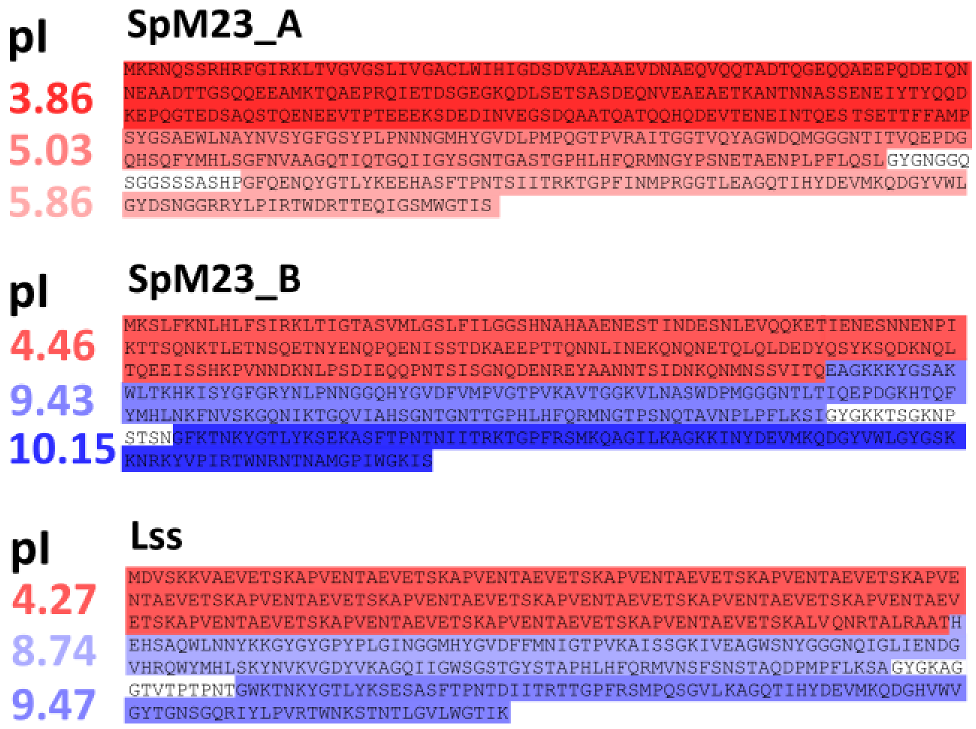

B.

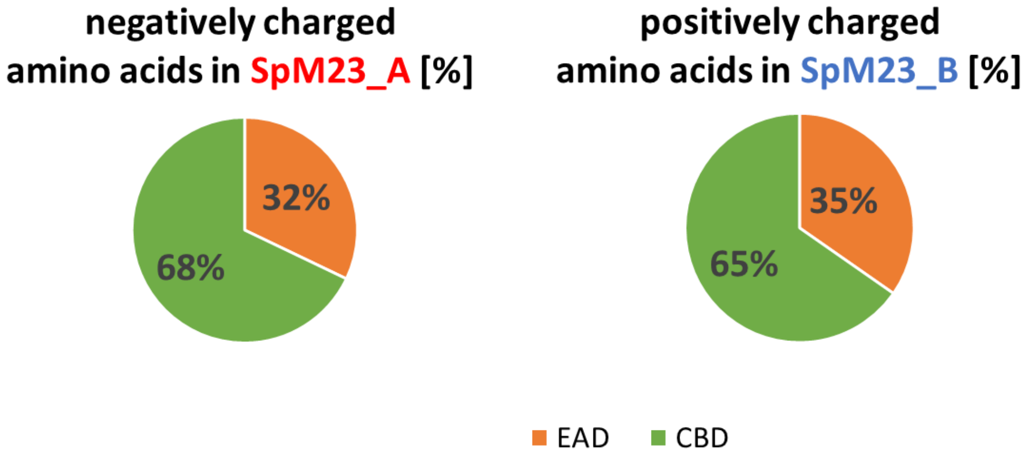

Figure S1. Charged amino acids distribution in catalytic and cell wall binding domains.

A. Residues encompassing domains of SpM23 enzymes and Lss.

B. The % values represent the negatively charged amino acids (aspartic and glutamic acid) in SpM23_A and positively charged amino acids(arginine and lysine) in SpM23_B.

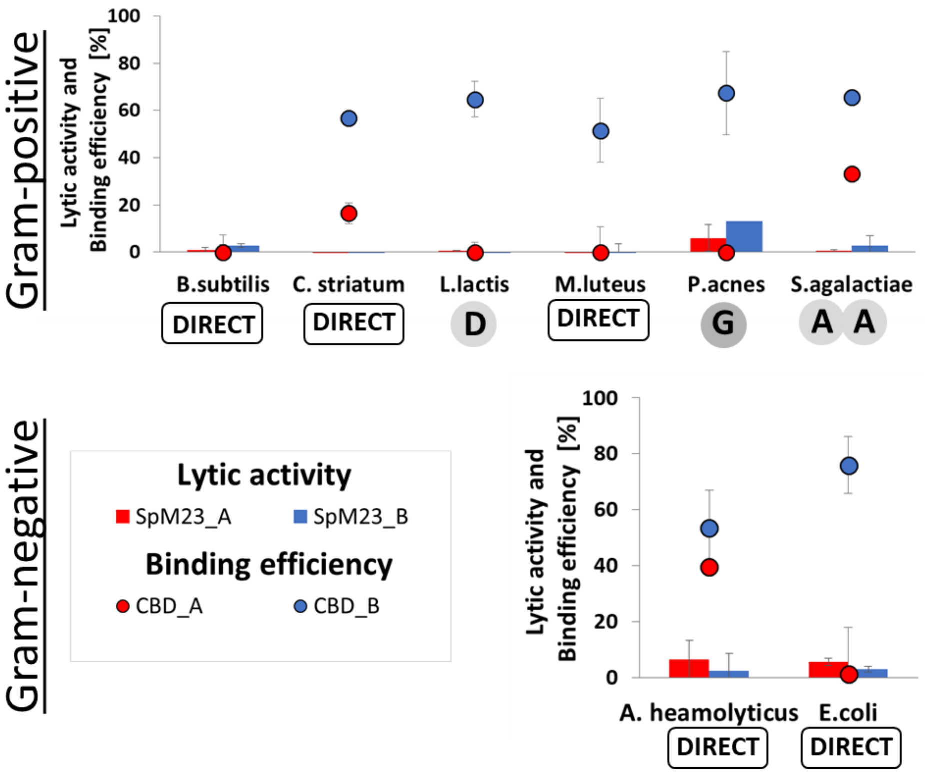

Figure S2. The lytic activity of SpM23_A and SpM23_B against non-staphylococcal strains. The lytic activity and binding spectra of the SpM23_A and SpM23_B and their CBDs. The results of the lytic activity assay are indicated with chart bars. The lytic activity was tested in a turbidity reaction assay performed in 50 mM glycine pH 8.0 and 100 mM NaCl buffer in the presence of 100 nM enzymes for 1 h at room temperature (RT). The results are shown as columns presenting the percentage of reduction of the initial turbidity of the bacterial cell suspensions, subtracted from the negative control that was incubated without enzymes. Abbreviations: *B. subtilis - Bacillus subtilis*; *C. striatum – Corynebacterium striatum*; *L. lactis – Lactobacillus lactis*; *M. luteus – Micrococcus luteus*; *P. acnes – Propionibacterium (Cutibacterium) acnes*; *S. agalactiae – Streptococcus agalactiae*; *A. haemolyticus – Acinetobacter haemolyticus*. The error bars represent the standard deviation, calculated in three independent assays. Cross-bridge amino acid composition was indicated in grey in single letter code.

The bindings of CBD_A and CBD_B are shown as dots. The bacterial cells were mixed with 1 µM each fluorescently labeled CBD in PBS buffer pH 7.2, incubated at RT. The cells were pelleted, and the fluorescence of the unbound fraction in the supernatant was measured; the results were normalized to the fluorescence of negative control (GFP only). The results are presented as percentages of reduced fluorescence after the addition of the tested CBDs. Schematic representation of the cross-bridge composition has been presented below the bacterial names in the polarity from diamino acid side chain to D-Ala (listed as well in **Table S1A**).The error bars represent the standard deviation, calculated in three independent assays.

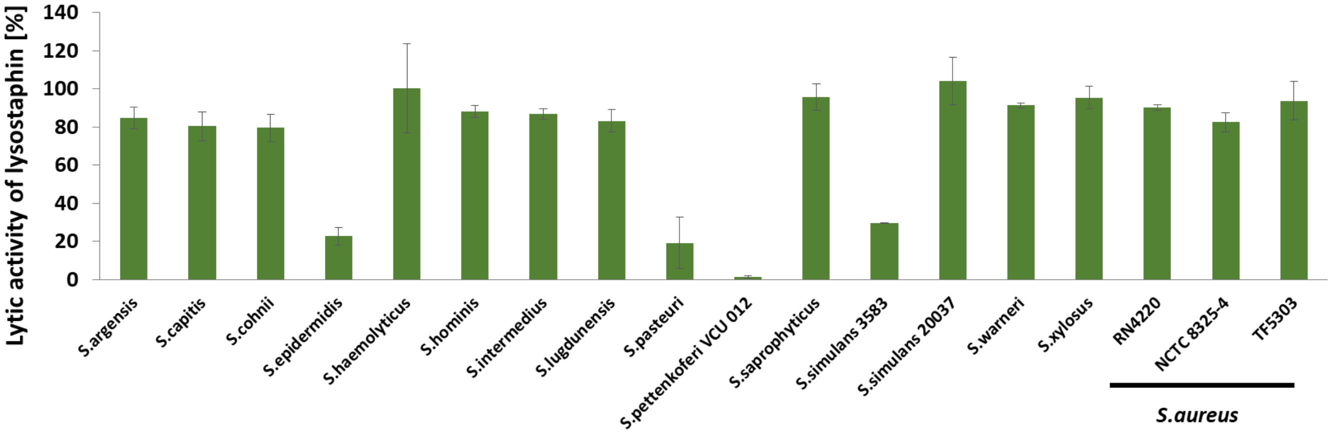

Figure S3. Lytic activity of the lysostaphin against staphylococci. Lytic activity is indicated with chart bars. The assay was performed in 50mM glycine pH 8.0 buffer with 100mM NaCl. The chart shows bacterial suspension turbidity reduction after 1 hour incubation with 100nM enzymes at RT. Result are presented in % relative to time point zero. The decrease in the turbidity of the bacteria suspension in the reaction buffer without addition of the lytic enzyme was treated as negative control and subtracted from the presented values. The experiment was performed three independent times.

A.

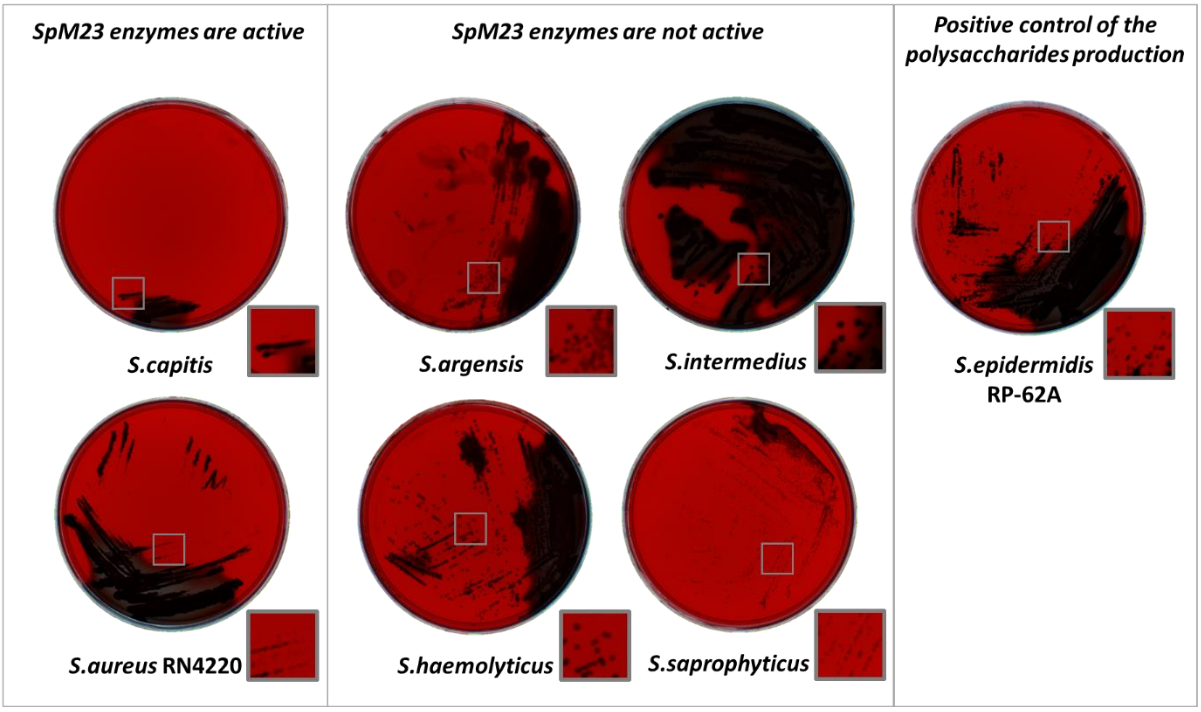

B.

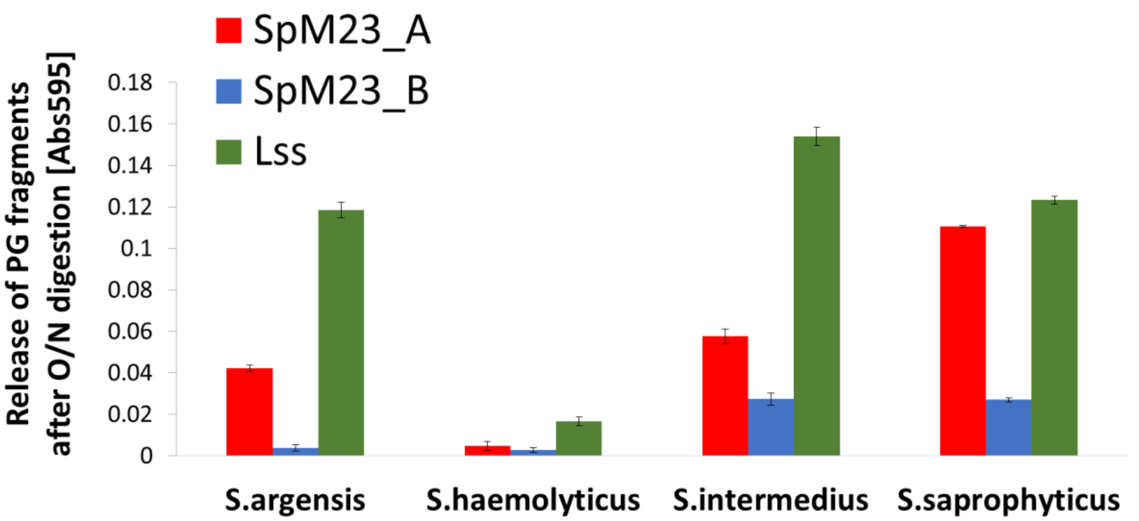

Figure S4. Determination of the factors that may cause decreased susceptibility of *S.argensis, S.haemolyticus, S.intermedius* and *S.saprophyticus* to SpM23 enzymes.

A. Expopolysaccharide production. Stains were spread on the Congo Red agar in order to study the exopolysaccharide production (11). The *S.epidermidis* RP-62A was treated as the positive control for their production (8). Plates were incubated overnight at 37°C.

B. Peptidoglycan composition. Activity of SpM23 enzymes against PG purified from four strains that display decreased susceptibility to SpM23 enzymes. The assay was performed in 50mM glycine pH 8.0, 100mM NaCl. The chart shows release of soluble fragments of PG stained with Remazol Brilliant Blue R after overnight incubation with 100 nM enzymes at 37°C. The increase in the absorbance in the sample without addition of the lytic enzyme was treated as negative control and subtracted from the presented values. Experiment was performed in technical triplicate.

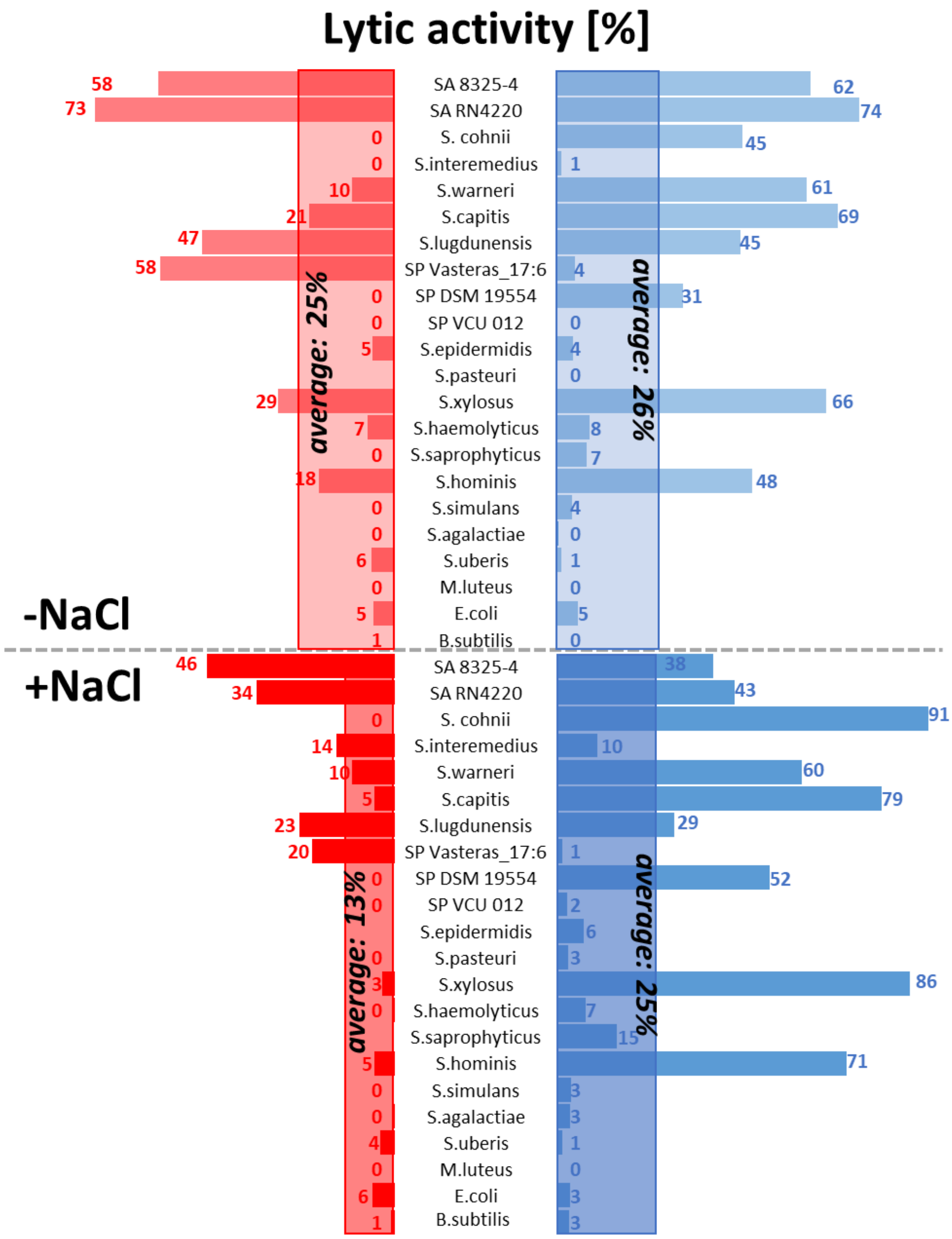

Figure S5. Lytic activity of the SpM23_A and SpM23_B in different ionic conditions. Lytic activity assay is indicated with chart bars and the average for each enzyme was depicted with colored rectangles. The assay was performed in 50mM glycine pH 8.0 buffer with 100mM NaCl ("+NaCl") or without ("-NaCl"). The chart shows bacterial suspension turbidity reduction after 1 hour incubation with 100nM enzymes at RT. Result are presented in % relative to time point zero. The decrease in the turbidity of the bacteria suspension in the reaction buffer without addition of the lytic enzyme was treated as negative control and subtracted from the presented values. Abbreviations: SA – *Staphylococcus aureus,* SP – *Staphylococcus pettenkoferi, B. subtilis* –belong to *Bacillus* genus*, S.agalactiae* – to *Streptococcus, S.simulans* - *S.simulans* 3583. The experiment was performed three independent times.

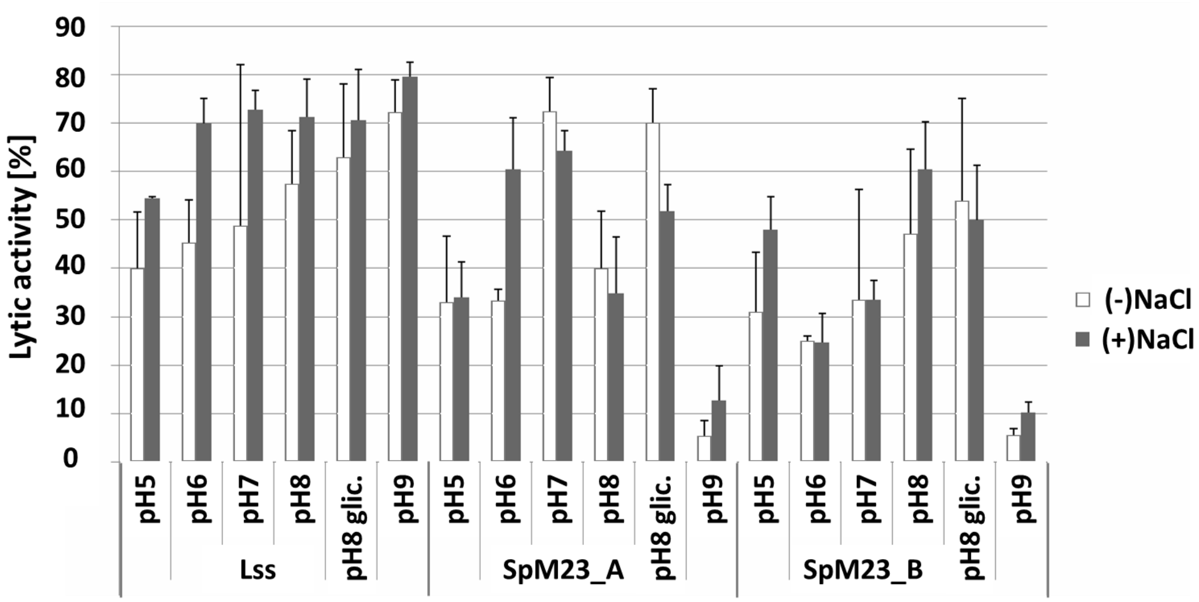

Figure S6. Lytic activity of the SpM23_A and SpM23_B across the spectrum of buffers of different pH. The assay was performed against *S. aureus* NCTC 8325-4. The assay was performed in different buffers (see: Materials and Methods) of the same conductivity (0.5 mS/cm), supplemented with 100mM NaCl ("+NaCl") or not ("-NaCl"). The chart shows bacterial suspension turbidity reduction after 1 hour incubation with 100 nM enzymes at RT. Result are presented in % relative to time point zero. The decrease in the turbidity of the bacteria suspension in the reaction buffer without addition of the lytic enzyme was treated as negative control and subtracted from the presented values. Abbreviations: glic. - 50 mM glycine buffer pH 8.0. The experiment was performed three independent times. The standard deviation is indicated with a bars.

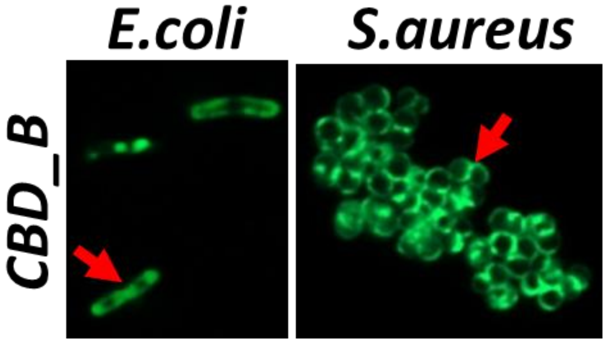

Figure S7. Imaging of *S. aureus* RN4220 (ΔtagO mutant) and *E. coli* BL21 stained with CBD_B fused to GFP. The overnight culture of bacterial cells resuspended in PBS pH 7.2 and CBD_B fused to GFP was added. After short incubation, the cells were centrifuged and resuspended in fresh portion of PBS. The obtained sample was subjected to imaging by confocal microscope (LSM 800, Zeiss). The CBD_B binding to division septa was indicated with red arrows.

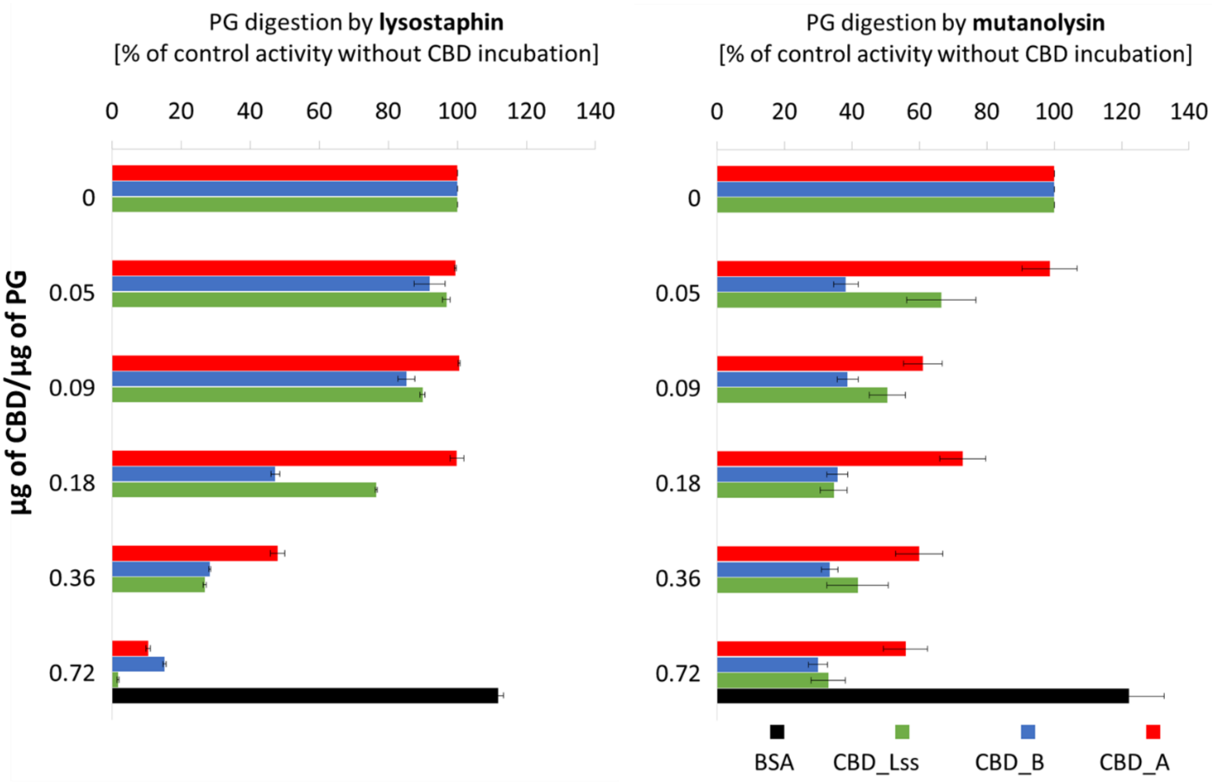

Figure S8. Competitive assay of the CBDs with Lss (left panel) and mutanolysin (right panel) performed on the PG purified from *S.aureus* NCTC 8325-4 and *S.agalactiae*.

The assay was performed in 50mM glycine pH 8.0, 100mM NaCl. The chart shows release of soluble fragments of PG stained with Remazol Brilliant Blue R after 1 hour incubation with 100 nm of Lss and or after 2 h incubation of 100 U of mutanolysin at 37°C. The increase in the absorbance in the sample without addition of the lytic enzyme was treated as negative control and subtracted from the presented values. Experiment was performed in technical triplicate. BSA was used as a negative control to identify the unspecific binding to PG in the studied reaction conditions.

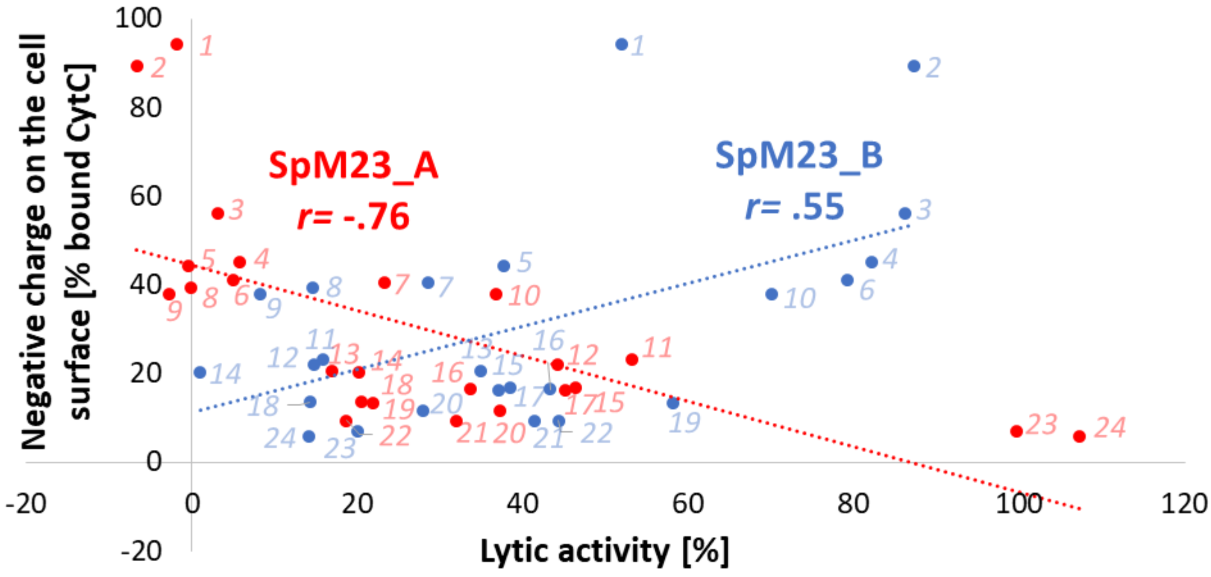

ID	Species	ID	Species
1	*S.pettenkoferi* DSM19554	13	*S.aureus* 8325
2	*S.cohnii*	14	*S.pettenkoferi* 17:6
3	*S.xylosus*	15	*S.aureus* 8325-4
4	*S.aureus* RN4220 ΔltaS	16	*S.aureus* RN4220
5	*S.argensis*	17	*S.pettenkoferi* 18:1
6	*S.capitis*	18	*S.pettenkoferi* 17:2
7	*S.lugdunensis*	19	*S.aureus* PS187
8	*S.saprophyticus*	20	*S.aureus* RN4220 ΔMΔS
9	*S.epidermidis*	21	*S.aureus* RN4220 ΔS
10	*S.aureus* 8325 ΔdltA	22	*S.aureus* RN4220 ΔM
11	*S.pettenkoferi* 17:8	23	*S.aureus* PS187 ΔtagO
12	*S.pettenkoferi* 17:5	24	*S.aureus* RN4220 ΔtagO

Figure S9. The trend line and Pearson coefficient (r) for the correlation between the negative surface charge of *Staphylococcus* spp. envelope presented as the % of bound cytochrome C and the mean lytic enzyme activity in turbidity reduction assay. Correlation analysis was done for *Staphylococcus* spp., since the lytic activity of SpM23 enzymes was limited to this particular genus, thus contain correct PG substrate. The lytic activity values indicated the OD_{600} decrease after 1h of reaction. The bacteria suspended in the reaction buffer without addition of the lytic enzyme were treated as negative control and the OD_{600} decrease of such control were subtracted from the presented values. The reaction buffer was 50mM glycine buffer pH 8.0, 100 mM NaCl. The CytC binding is performed in the octane buffer pH 4.6. All experiments were performed 3 times. The data dots, trend line and Pearson coefficient (r) was coloured according to each studied data set, red being the correlation with SpM23_A activity and blue, with SpM23_B. The ID numbers indicated on the chart correspond to the species names listed in table below the chart.

A.

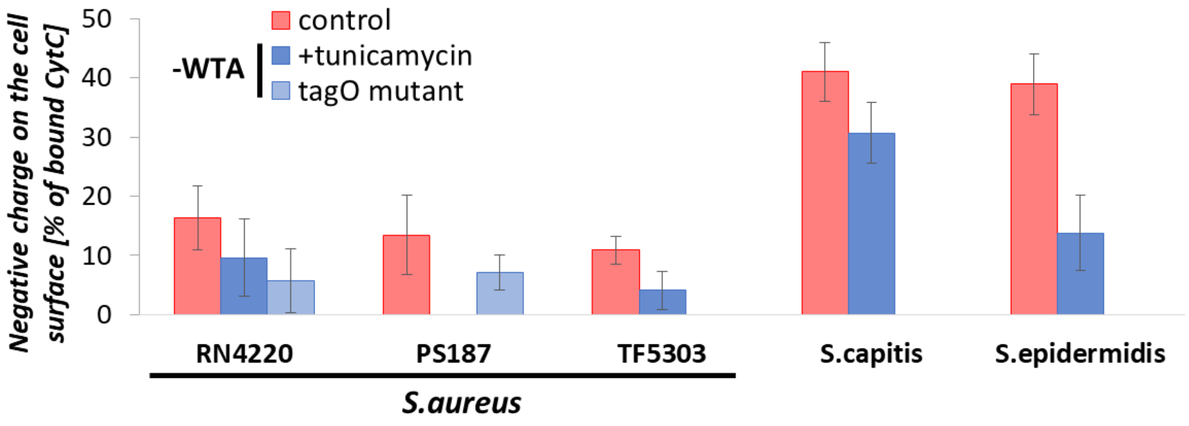
Negative charge on the cell surface [% of bound CytC]
50
40
30
20
10
0
control
-WTA
+tunicamycin
tagO mutant
RN4220
PS187
TF5303
S.aureus
S.capitis
S.epidermidis

B.

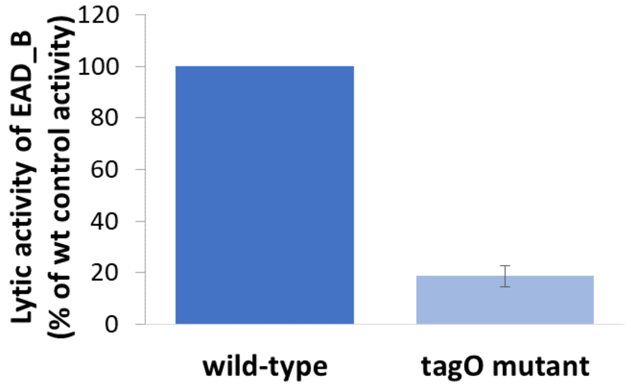
Lytic activity of EAD_B (% of wt control activity)
120
100
80
60
40
20
0
wild-type
tagO mutant

C.

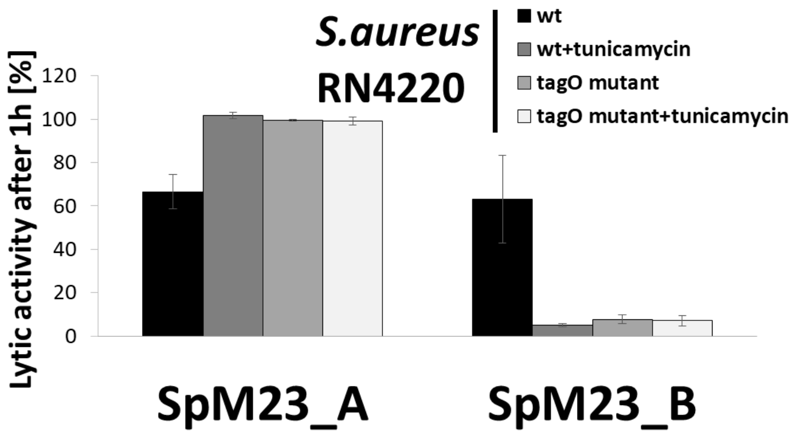
S.aureus RN4220
wt
wt+tunicamycin
tagO mutant
tagO mutant+tunicamycin
Lytic activity after 1h [%]
120
100
80
60
40
20
0
SpM23_A
SpM23_B

D.

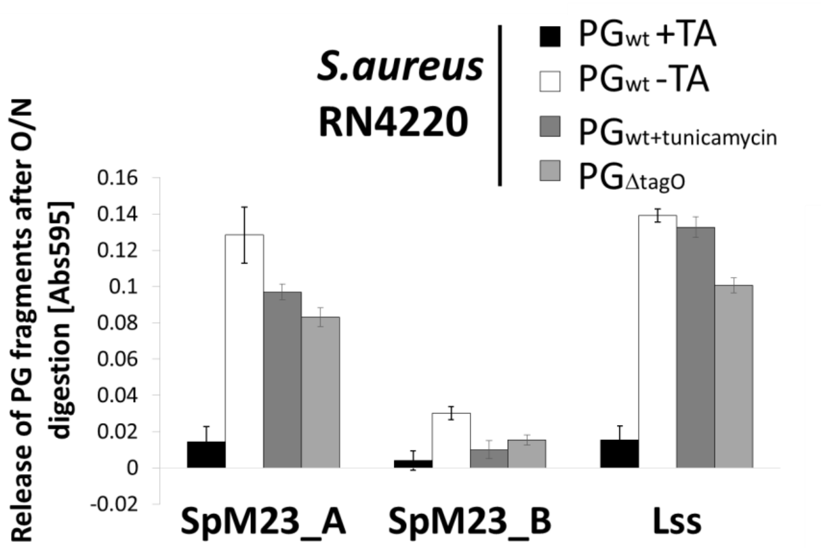
S.aureus
RN4220
PGwt +TA
PGwt -TA
PGwt+tunicamycin
PGΔtagO
Release of PG fragments after O/N digestion [Abs595]
0.16
0.14
0.12
0.1
0.08
0.06
0.04
0.02
0
-0.02
SpM23_A
SpM23_B
Lss

E.

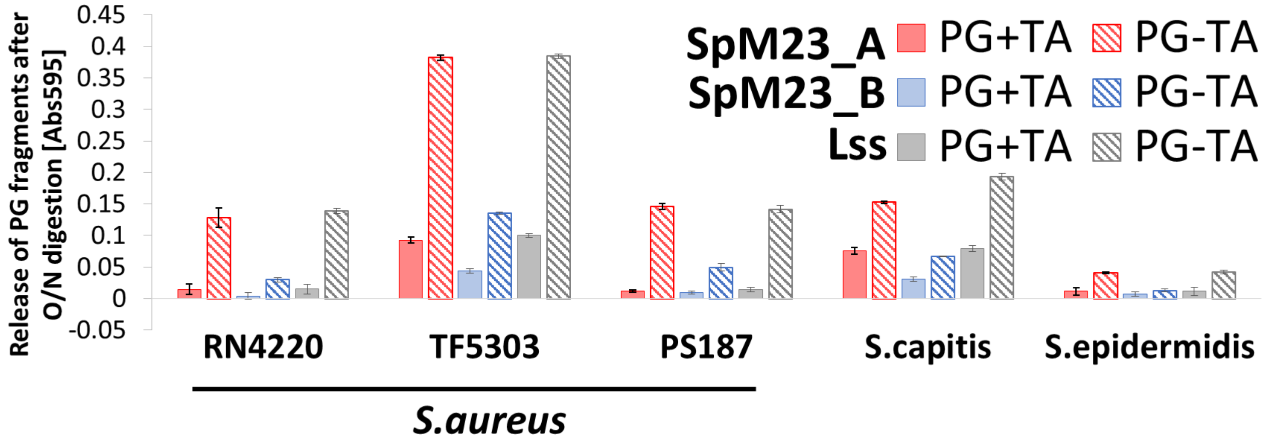
Release of PG fragments after O/N digestion [Abs595]
0.45
0.4
0.35
0.3
0.25
0.2
0.15
0.1
0.05
0
-0.05
SpM23_A PG+TA PG-TA
SpM23_B PG+TA PG-TA
Lss PG+TA PG-TA
RN4220
TF5303
PS187
S.capitis
S.epidermidis
S.aureus

F.

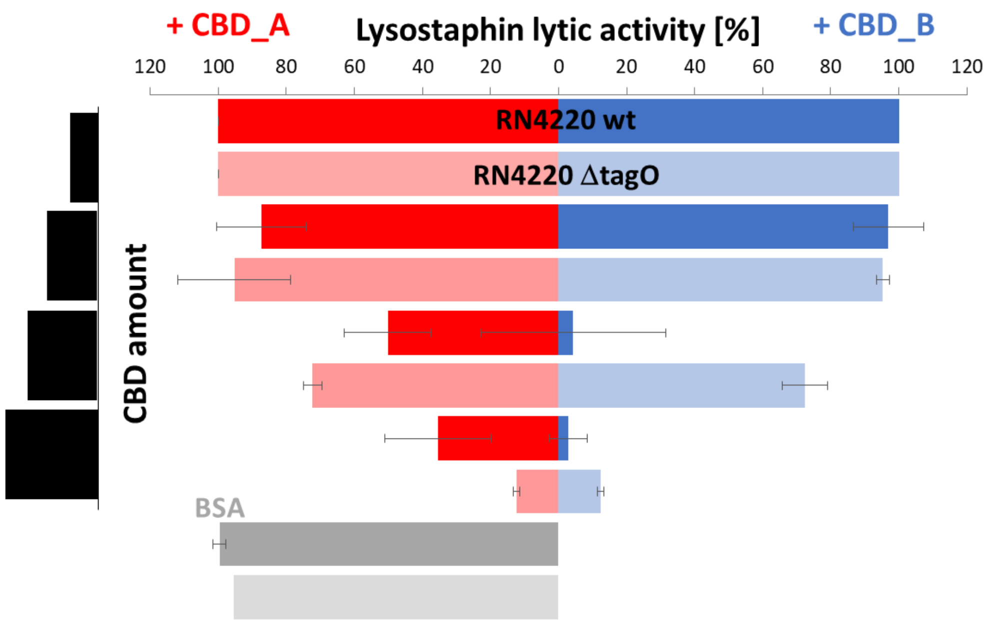

G.

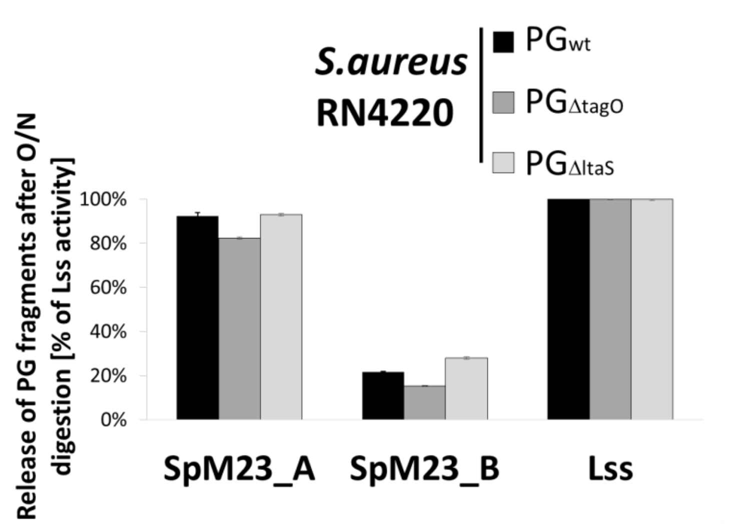

Figure S10. The effects of WTA depletion.

A. Surface charge of selected bacterial species upon WTA depletion, estimated in CytC assay. The CytC binding is performed in the ammonium acetate buffer pH 4.6. The values obtained in the CytC assay were coloured accordingly to the code, where the one of most negative charge is marked in red and the most positive in blue. Values were related to 100%. The experiments were performed 3 independent times and each bar represents the average, error bars represent standard deviation.

B. Lytic activity of SpM23_B enzymatically active domain. The assay was performed in 50mM glycine pH 8.0. The chart shows bacterial turbidity reduction after 1 hour incubation with 500 nM enzymes at RT. Result are presented in relative to wild-type. The decrease in the turbidity of the bacteria

suspension in the reaction buffer without addition of the lytic enzyme was treated as negative control and subtracted from the presented values.

C. Lytic activity of SpM23 enzymes against *S.aureus* RN4220 wild-type and tagO mutant and the effect of tunicamycin on their action. The assay was performed in 50mM glycine pH 8.0. The chart shows bacterial turbidity reduction after 1 hour incubation with 100 nM enzymes at RT. The decrease in the turbidity of the bacteria suspension in the reaction buffer without addition of the lytic enzyme was treated as negative control and subtracted from the presented values.

D. Activity of SpM23 enzymes against PG purified from *S.aureus* RN4220 wild-type +/-TA (removed by trichloroacetic acid [TCA] treatment), *S.aureus* RN4220 wild-type cultured in the presence of tunicamycin and *S.aureus* RN4220 tagO mutant. The assay was performed in 50mM glycine pH 8.0, 100mM NaCl. The chart shows release of soluble fragments of PG stained with Remazol Brilliant Blue R after overnight incubation with 100 nM enzymes at 37°C. The increase in the absorbance in the sample without addition of the lytic enzyme was treated as negative control and subtracted from the presented values. Experiment was performed in technical triplicate.

E. Activity of SpM23 enzymes against PG purified from the same set of strains that were treated with tunicamycin (**Figure 7A**). EACH PG sample was compared with its counterpart depleted of TA upon TCA treatment. Conditions of the reaction were the same as described in panel **D**.

F. Competition assay with lysostaphin and two cell wall binding domains against *S.aureus* RN4220 (regular colors) and RN4220 ΔtagO mutant (pale colors). Results are shown relative to the sample without addition of the binding domain. The concentration of the highest blocking amount of CBD_A and CBD_B was used to perform experiment with BSA and treat it as a negative control.

G. Activity of SpM23 enzymes against PG purified from *S.aureus* RN4220 wild-type, tagO mutant and ltaS mutant. Presented values were normalized to the activity of Lss that was treated as an internal control, to avoid discrepancies resulting from the different staining efficiency between PG samples. Conditions of the reaction were the same as described in panel **D**.

A.

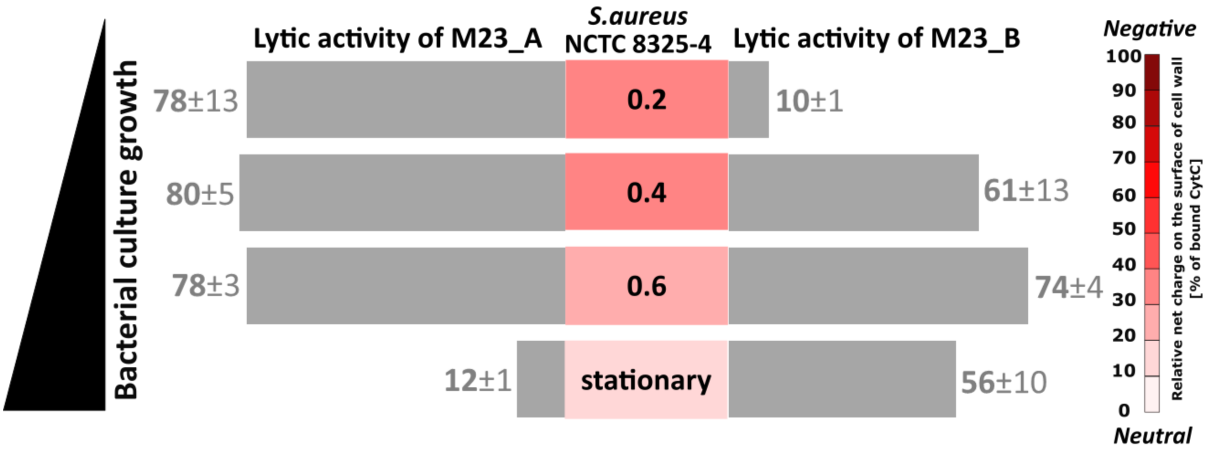

B.

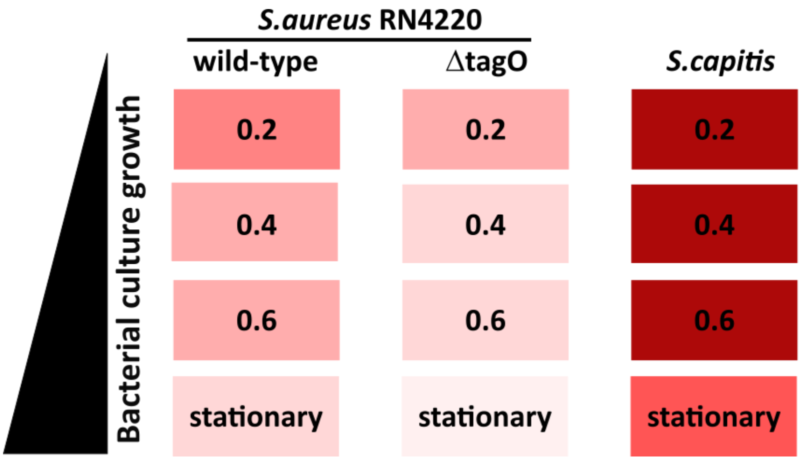

Figure S11. The bacterial net charge alterations in the curse of bacterial culture growth.

A. The activity of SpM23_A and SpM23_B enzymes against *S.aureus* NCTC 8325-4C bacteria collected at various phase of the culture growth. The lytic activity tests were performed in 50mM glycine buffer pH 8.0. The chart shows bacterial turbidity reduction after 1 hour incubation with 100 nM enzymes at RT. Result are presented in relative to time-point zero. The decrease in the turbidity of the bacteria suspension in the reaction buffer without addition of the lytic enzyme was treated as negative control and subtracted from the presented values.

B. Alternations of the bacterial net charge of *Staphylococcus* spp.

The surface net charge of the presented strains was estimated with CytC assay the same way as described in Figure 6A.

References:

1. Gross M, Cramton SE, Götz F, Peschel A. 2001. Key role of teichoic acid net charge in Staphylococcus aureus colonization of artificial surfaces. Infect Immun 69:3423–3426.
2. Sugai M, Fujiwara T, Ohta K, Komatsuzawa H, Ohara M, Suginaka H. 1997. epr, which encodes glycylglycine endopeptidase resistance, is homologous to femAB and affects serine content of peptidoglycan cross bridges in Staphylococcus capitis and Staphylococcus aureus. J Bacteriol 179:4311–4318.
3. Hübscher J, Jansen A, Kotte O, Schäfer J, Majcherczyk PA, Harris LG, Bierbaum G, Heinemann M, Berger-Bächi B. 2007. Living with an imperfect cell wall: compensation of femAB inactivation in Staphylococcus aureus. BMC Genomics 8:307.
4. Weidenmaier C, Kokai-Kun JF, Kristian SA, Chanturiya T, Kalbacher H, Gross M, Nicholson G, Neumeister B, Mond JJ, Peschel A. 2004. Role of teichoic acids in Staphylococcus aureus nasal colonization, a major risk factor in nosocomial infections. Nat Med 10:243–245.
5. Winstel V, Liang C, Sanchez-Carballo P, Steglich M, Munar M, Bröker BM, Penadés JR, Nübel U, Holst O, Dandekar T, Peschel A, Xia G. 2013. Wall teichoic acid structure governs horizontal gene transfer between major bacterial pathogens. Nat Commun 4:2345.
6. Gründling A, Schneewind O. 2007. Synthesis of glycerol phosphate lipoteichoic acid in Staphylococcus aureus. Proc Natl Acad Sci U S A 104:8478–8483.
7. Winstel V, Sanchez-Carballo P, Holst O, Xia G, Peschel A. 2014. Biosynthesis of the unique wall teichoic acid of Staphylococcus aureus lineage ST395. mBio 5:e00869.
8. Tojo M, Yamashita N, Goldmann DA, Pier GB. 1988. Isolation and characterization of a capsular polysaccharide adhesin from Staphylococcus epidermidis. J Infect Dis 157:713–722.
9. Månsson E, Hellmark B, Stegger M, Skytt Andersen P, Sundqvist M, Söderquist B. 2017. Genomic relatedness of Staphylococcus pettenkoferi isolates of different origins. J Med Microbiol 66:601–608.
10. Kozlowski LP. 2022. Proteome-pI 2.0: proteome isoelectric point database update. Nucleic Acids Res 50:D1535–D1540.
11. Freeman DJ, Falkiner FR, Keane CT. 1989. New method for detecting slime production by coagulase negative staphylococci. J Clin Pathol 42:872–874.
